# Etk/Bmx Regulates Proteinase-Activated-Receptor1 (PAR_1_) in Breast Cancer Invasion: Signaling Partners, Hierarchy and Physiological Significance

**DOI:** 10.1371/journal.pone.0011135

**Published:** 2010-06-15

**Authors:** Irit Cohen, Myriam Maoz, Hagit Turm, Sorina Grisaru-Granovsky, Bella Maly, Beatrice Uziely, Einat Weiss, Rinat Abramovitch, Eithan Gross, Oded Barzilay, Yun Qiu, Rachel Bar-Shavit

**Affiliations:** 1 Department of Oncology, Hadassah-University Hospital, Jerusalem, Israel; 2 Department of Pathology, Hadassah-University Hospital, Jerusalem, Israel; 3 Goldyne Savad Institute for Gene Therapy, Hadassah-University Hospital, Jerusalem, Israel; 4 Department of Pediatric Surgery, Hadassah-University Hospital, Jerusalem, Israel; 5 Department of Pharmacology and Experimental Therapeutics, University of Maryland School of Medicine, Baltimore, Maryland, United States of America; Baylor College of Medicine, United States of America

## Abstract

**Background:**

While *protease-activated-receptor 1* (PAR_1_) plays a central role in tumor progression, little is known about the cell signaling involved.

**Methodology/Principal Findings:**

We show here the impact of PAR_1_ cellular activities using both an orthotopic mouse mammary xenograft and a colorectal-liver metastasis model *in vivo*, with biochemical analyses *in vitro*. Large and highly vascularized tumors were generated by cells over-expressing *wt hPar1*, *Y397Z hPar1*, with persistent signaling, or *Y381A hPar1* mutant constructs. In contrast, cells over-expressing the truncated form of *hPar1*, which lacks the cytoplasmic tail, developed small or no tumors, similar to cells expressing empty vector or control untreated cells. Antibody array membranes revealed essential *hPar1* partners including Etk/Bmx and Shc. PAR_1_ activation induces Etk/Bmx and Shc binding to the receptor C-tail to form a complex. Y/A mutations in the PAR_1_ C-tail did not prevent Shc-PAR_1_ association, but enhanced the number of liver metastases compared with the already increased metastases obtained with *wt hPar1*. We found that Etk/Bmx first binds via the PH domain to a region of seven residues, located between C378-S384 in PAR_1_ C-tail, enabling subsequent Shc association. Importantly, expression of the *hPar1*-7A mutant form (substituted A, residues 378-384), which is incapable of binding Etk/Bmx, resulted in inhibition of invasion through Matrigel-coated membranes. Similarly, knocking down Etk/Bmx inhibited PAR_1_-induced MDA-MB-435 cell migration. In addition, intact spheroid morphogenesis of MCF10A cells is markedly disrupted by the ectopic expression of *wt hPar1*. In contrast, the forced expression of the *hPar1-*7A mutant results in normal ball-shaped spheroids. Thus, by preventing binding of Etk/Bmx to PAR_1_ -C-tail, *hPar1* oncogenic properties are abrogated.

**Conclusions/Significance:**

This is the first demonstration that a cytoplasmic portion of the PAR_1_ C-tail functions as a scaffold site. We identify here essential signaling partners, determine the hierarchy of binding and provide a platform for therapeutic vehicles via definition of the critical PAR_1_ **-**associating region in the breast cancer signaling niche.

## Introduction

*Protease-activated receptor-1* (PAR_1_), a G protein-coupled receptor (GPCR), is the first and prototype member of the mammalian PAR family, which comprises four genes. The activation of PAR_1_ involves the release of an N-terminal peptide and the exposure of an otherwise hindered ligand, resulting in an exclusive mode of activation and a general paradigm for the entire PAR family **(1–3).** While a well-known classical observation points to a tight link between hyper-activation of the coagulation system and cancer malignancies, the molecular mechanism that governs pro-coagulant tumor progression remains poorly defined [**1]**, [2], [3****]. Surprisingly, the zinc-dependent matrix-metalloprotease 1 (MMP-1), a collagenase that efficiently cleaves extracellular matrix (ECM) and basement membrane components, has been shown to specifically activate PAR_1_ [4]. The PAR_1_ -MMP1 axis may thus provide a direct mechanistic link between PAR_1_ and tumor metastasis.

Levels of *hPar1* expression and epithelial tumor progression are correlated in both clinically obtained biopsy specimens and a wide spectrum of differentially metastatic cell lines [5], [6]. PAR_1_ also plays a role in the physiological invasion process of placental cytotrophoblasts during implantation into the uterus decidua [7]. Trophoblast invasion shares many features with the tumor cell invasion process; it differs, however, by the time-limited *hPar1* expression, which is confined to the trophoblast-invasive period, and is shut off immediately thereafter, when the need to invade disappears [7]. This provides strong support for the idea that the *hPar1* gene is part of an invasive gene program.

Importantly, PAR_1_ cellular trafficking and signal termination appear to occur in a different mode than other GPCRs. Instead of recycling back to the cell surface after ligand stimulation, activated PAR_1_ is sorted to the lysosomes and degraded [8], [9]. Aberrant PAR_1_ trafficking, resulting in receptor-populated cell surfaces and causing prolonged and persistent signals, has been found in breast cancer [10]. While cellular trafficking of PAR_1_ impinges on the extent and mode of signaling, identification of individual PAR_1_ signaling partners and their contribution to breast cancer progression remain to be elucidated.

In the present study, we have identified PAR_1_ C-tail as a scaffold site for the immobilization of signaling partners. In addition to identifying key partners, we have determined the hierarchy of binding and established a region in PAR_1_ C-tail critical for breast cancer signaling. The association of Etk/Bmx and Shc to form a physical complex with PAR_1_ C-tail is demonstrated. The prime link of Etk/Bmx to PAR_1_ is mediated via its PH domain, enabling the subsequent immobilization of Shc. The physiological significance of PAR_1_-Etk/Bmx binding is emphasized by the inhibition of Matrigel invasion and appearance of nearly intact acini morphogenesis of polarized cell architecture when this site is mutated. The use of consecutive A residues inserted into the proposed Etk/Bmx binding region of PAR_1_ C-tail (e.g., *hPar1*-7A) abolished PAR_1_ -induced pro-oncogenic properties. Thus, by preventing the binding of a key signaling partner to PAR_1_ C-tail, efficient inhibition of PAR_1_ -induced tumor-associated functions, including loss of epithelial cell polarity, migration and invasion through basement membranes, is obtained. Elucidation of the PAR_1_ C-tail binding domain may provide a platform for new therapeutic vehicles in treating breast cancer.

## Results

### PAR_1_-enhanced tumor growth and angiogenesis *in vivo* is abrogated in the presence of a truncated PAR_1_ form

**Figure 1:**
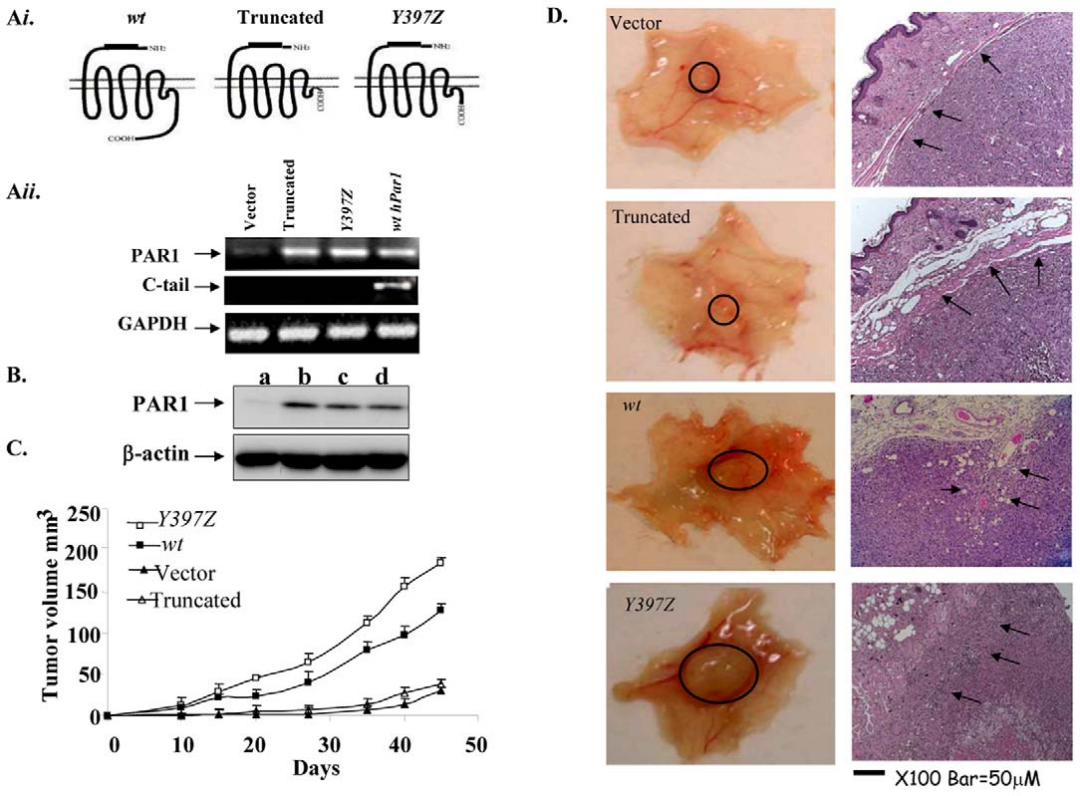
PAR1 enhances tumor growth and angiogenesis in a xenograft mouse model. **A** *i. Schematic representation of the hPar1 constructs. wt hPar1*, truncated *hPar1* (devoid of the cytoplasmic tail), *Y397Z hPar1* (shorter C-tail of persistent signaling). **A** *ii. Semi-quantitative RT-PCR analysis of various hPar1 constructs.* RT-PCR analysis of cells transfected with *wt hPar1*, *Y397Z hPar1*, truncated *hPar1* or empty vector using primers to PAR_1_ N-terminus (upper panel), C-terminus (middle panel), or GAPDH (lower panel). PAR_1_ N-terminus primers were as follows: *hPar1*-sense: 5′- CTCGTCCTCAAGGAGCAAAC-3′, antisense orientation: 5′-TGGGATCGGAACTTTCTTTG-3' (564-bp PCR product). PAR_1_ C-tail primers – sense: 5′-TAC TAT TAC GCT GGA TCC TCT GAG-3′ and antisense: 5′-CTT GAA TTC CTA AGT TAA CAGCTT-3′. These primers give rise to a 181-bp product corresponding to the entire PAR_1_ C-tail site, as follows: YY – YASSECQRYVYSILCCKESSDPSYNSSGQLMASKMDTCSSNLNNSIYKKLLT. **B**. *Western blot analyses of MCF7 cells expressing various hPar1 constructs.* a. Mock-transfected MCF7; b. *Y397Z hPar1*; c. Truncated *hPar1*; d. *wt hPar1*. **C.** *Mouse mammary tumor growth in animals implanted with cells expressing wt hPar1 and variants.* MCF7 cells expressing various *hPar1* were inoculated into the mammary pads of mice. Tumor volumes (mean ± SD) are shown for *wt hPar1* (▪), *Y397Z hPar1* (□), truncated *hPar1* (Δ), and empty vector (▴; tumor volume 30±3 mm^3^)(* *P<*0.005). **D**. *Morphological appearance.* The *Y397Z hPar1* and *wt hPar1* constructs exhibited intense vascularization and appeared reddish as opposed to the pale appearance of tumors generated by either empty vector or truncated *hPar1*. Tissue sections of tumors were stained with H&E (right panels). Magnification is ×100.

**Figure 2:**
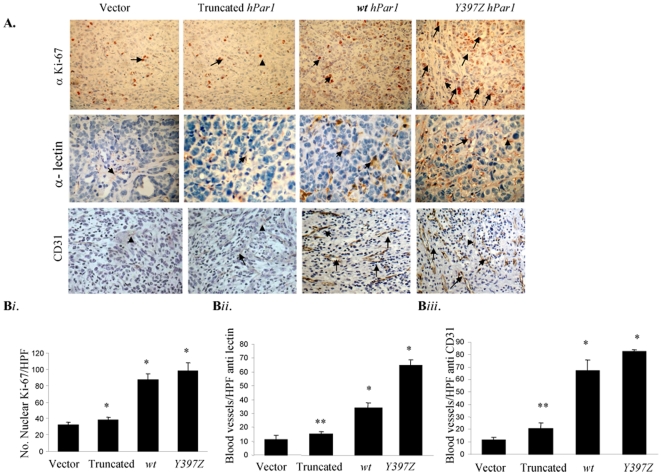
Proliferation and blood vessel formation in tumors produced by *hPar1* constructs. **A**. Sections of tumors generated by various *hPar1* constructs were subjected to immunohistochemical staining with Ki-67 for proliferation (upper panel), and with either an endothelial cell-specific lectin or anti-CD31 to visualize blood vessels (lower panel). **B** *i*. Ki-67-positive cells were counted in five microscope fields per tumor section, and the mean (± SD) number of cells per high power field (HPF) was determined. Significantly, fewer Ki-67-positive cells were observed in tumors produced by the empty vector or truncated *hPar1* constructs than with *wt hPar1* or *Y397Z hPar1*. **B** *ii* and *iii*. Anti-lectin- or anti-CD31-stained cells, respectively, were counted as described in (B*i*). The morphometric analysis used to evaluate proliferation and blood vessels was performed as explained in Materials& Methods under the section of histological evaluation and scoring. Briefly, the microscope was calibrated with a micrometer slide before each measurement. Five microscopic fields were screened, 10 cells/field were selected and no less than 50 cells/tumor case were assessed. Error bars show +/− SD of mean and the *P* value was determined (** *P*<0.005 * *P*<0.001; Chi- square test). The data are representative of four independent experiments performed in triplicates.

To investigate the role of PAR_1_ signaling in breast tumor growth and vascularization *in vivo*, we over-expressed *wt hPar1* and deletion constructs [e.g., *L369Z*, which lacks the entire cytoplasmic tail, and *Y397Z*, which exhibits persistent signaling due to impaired internalization [11], [12]] in MCF7 cells. The functional outcome of MCF7 cells over-expressing various *hPar1* constructs *in vivo* was assessed by orthotopic mammary fat pad tumor development (proper expression and characterization of the plasmids are shown in Figure S1). MCF7 cells over-expressing either *Y397Z* or *wt hPar1* constructs (e.g., MCF7/*Y397Z hPar1*; MCF7/*wt hPar1*) markedly enhanced tumor growth *in vivo* following implantation into the mammary glands (Fig. 1C and D), whereas MCF7 cells over-expressing truncated *hPar1* behaved similar to control MCF7 cells in vector-injected mice, which developed only very small tumors (Fig. 1C). The tumors obtained with MCF7/*wt hPar1* and MCF7/*Y397Z hPar1* were 5 and 5.8 times larger, respectively, than tumors produced by the MCF7/empty vector-transfected cells. Histological examination (H&E staining) showed that while both MCF7/*wt hPar1* and MCF7/*Y397Z hPar1* tumors infiltrated into the fat pad tissues of the breast, the MCF7/*Y397Z hPar1* tumors further infiltrated the abdominal muscle (Fig. 1D). In contrast, tumors produced by empty vector or truncated *hPar1*-transfected cells were capsulated, with no obvious cell invasion. Proliferation levels were evaluated by immunostaining with Ki-67 and were 3 times higher in *Y397Z hPar1* or *wt hPar1* tumors (Fig. 2) than in the small tumors produced by either empty vector or truncated *hPar1*-transfected cells (p<0.0001, Fig. 2B). Tumor growth can also be attributed to blood vessel formation [13], [14]. The *hPar1*-induced breast tumor vascularization was assessed by immunostaining with anti-lectin- and anti-CD31 antibodies. Both MCF7/*Y397Z* and MCF7/*wt hPar1* tumors were intensely stained (Fig. 2A; B*ii* and *iii*). In contrast, only a few blood vessels were found in the small tumors of empty vector or truncated *hPar1* (Fig. 2A; B*ii* and *iii*). Thus, both MCF7/*wt hPar1* and MCF7/*Y397Z hPar1* cells were shown to effectively induce breast tumor growth, proliferation and angiogenesis, while the MCF7/truncated *hPar1* and MCF7/empty vector-expressing cells had no significant effect.

### PAR_1_ C-tail binds the Shc adaptor protein

**Figure 3:**
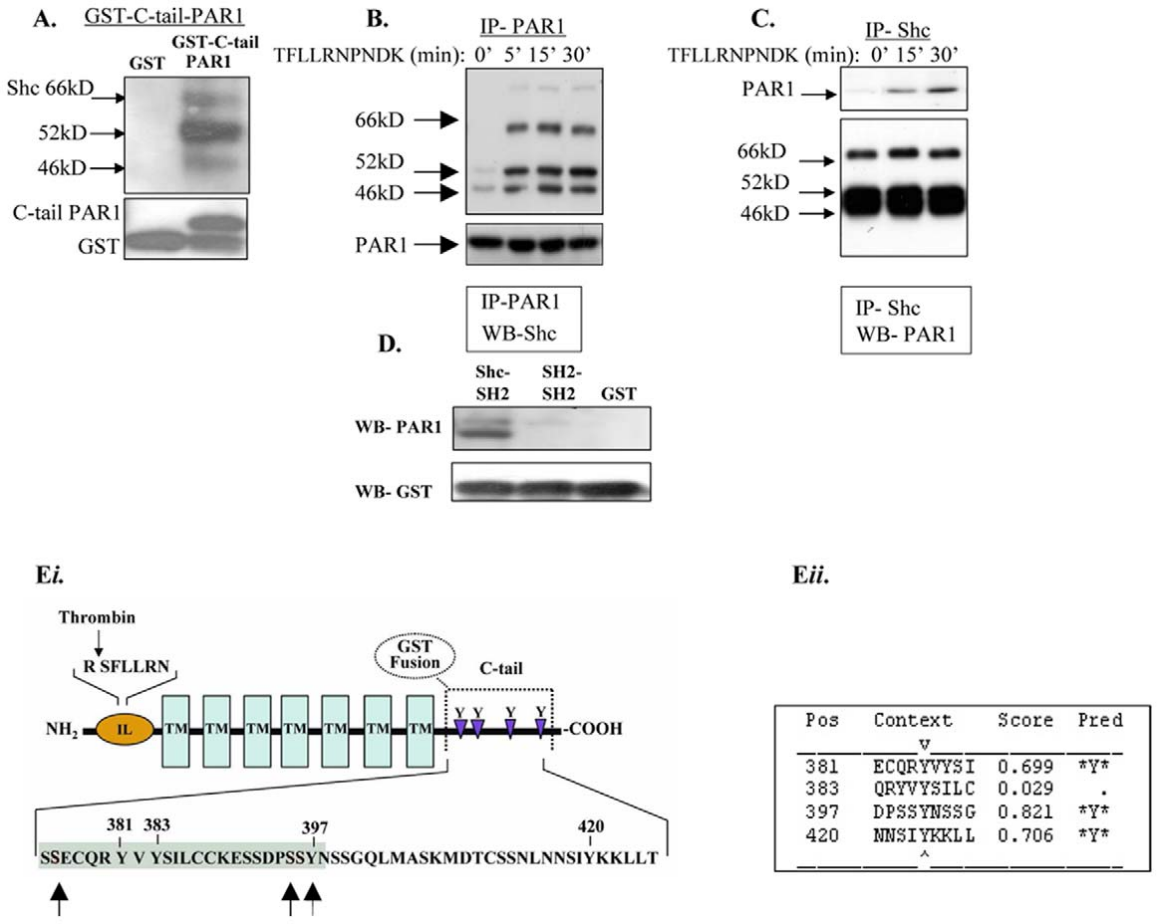
PAR_1_ C-tail recruits Shc adaptor protein. **A.** *PAR_1_ GST-C-tail binds Shc adaptor.* MDA-MB-435 cell lysates were applied to a GST- PAR_1_ C-tail or GST control column. After an adequate binding period of the designated cell lysates to the columns, a washing step was performed. This step was performed in order to wash out all non-specific proteins, leaving the GST- PAR_1_ -C-tail column firmly bound to targeted cell lysate proteins. Next, specifically-bound proteins were eluted via the addition of gel “sample buffer” and detected by western blot analysis using anti-Shc antibodies. **B** and **C**. *Co-immunoprecipitation analyses of PAR_1_ and Shc.* Lysates of non-activated or TFLLRNPNDK-activated MDA-MB-435 cells were co-immunoprecipitated with either anti- PAR_1_ (B) or anti-Shc (C) antibodies. **D**. *PAR_1_ binds to Shc-SH2 domain.* MDA-MB-435 cell lysates were loaded onto columns of GST-Shc-SH2, GST linked to a tandem SH2 from a non-relevant protein, or GST alone. Specifically-bound proteins were eluted and detected with anti- PAR_1_ antibodies. **E** ***i*** *. Schematic representation of PAR_1_ -C-tail*. The scheme illustrates PAR_1_ structure. IL - internal ligand, TM - transmembrane. Important tyrosine (Y) residues are indicated and the conserved sequence is gray. Serine residues in the C-tail region that may be involved in PAR_1_ -Shc association are indicated in red. **E** ***ii***. *Analysis of PAR_1_ C-tail Y-residues by NetPhos 2.0 server*. Y_381_, Y_397_ and Y_420_ were scored highly likely to undergo phosphorylation, as shown in the table. “Pred” means “prediction” for the predicted score of each of the Y tyrosine residues that is relevant to phosphorylation.

To identify proteins that associate with the PAR_1_ C-terminus and participate in the tumor signaling pathway, we fused the cytoplasmic tail of *hPar1* to a GST protein and used the construct as “bait” to specifically detect associated proteins. Lysates obtained from a highly metastatic breast carcinoma line (e.g., MDA-MB-435 cells) were assessed for binding to the GST- PAR_1_ C-tail column. Amino acid sequence analysis of proteins bound to the column repeatedly indicated the presence of the Shc adapter protein. Indeed, application of MDA-MB-435 cell lysates onto a GST- PAR_1_ C-tail column or a GST control column showed the three Shc isoforms specifically bound to the GST- PAR_1_ -C-tail column, but not to the GST control column (Fig. 3A). Shc isoforms refer to a series of proteins (e.g., 66, 52 and 46 kDa) termed Shc (Src homology 2/α-collagen–related) [15], [16]. cDNA analyses of the family proteins has demonstrated that the 46- and 52-kDa species arise from alternative translation initiation sites within the same transcript, giving rise to a 59-amino acid terminal truncation of the 46-kDa isoform compared to the 52-kDa isoform. In contrast, the 66-kDa species most likely arises from an alternatively spliced message since there is only one Shc gene and the carboxy terminal antibodies cross react with all three molecular weight species. Co-immunoprecipitation studies using either PAR_1_ (Fig. 3B) or Shc antibodies (Fig. 3C) confirmed the PAR_1_ -Shc association 5 min after TFLLRNPNDK activation; this association remained high during the 30 min of analysis (Fig. 3B and C). The Shc protein comprises multiple protein docking sites, including SH2, phospho-tyrosine binding site (PTB) and collagen homology domains 1 and 2 (CH1, CH2). When a GST-Shc-SH2 domain pull-down assay was used following loading with PAR_1_ activated MDA-MB-435 cell lysates, we obtained PAR_1_ -specific binding to the Shc-SH2 domain. In contrast, when the tandem SH2 domain from an irrelevant protein was used as a control, no binding of PAR_1_ was observed (Fig. 3D).

While searching for the PAR_1_ C-tail putative tyrosine residues capable of undergoing phosphorylation and serving as possible binding sites for the Shc protein (*NetPhos 2.0 server*), we found four candidates: Y_381_, Y_383_, Y_397_, Y_420_ (Fig. 3E *i* and *ii*). Of these, only three were predicted to undergo phosphorylation: Y_381_, Y_397_ and Y_420_ (Fig. 3E *ii*). Since our preliminary data showed that *Y397Z hPar1* was potent in signaling (Figs 1 and 2) and able to associate with Shc when transiently expressed in COS-1 cells (data not shown), we postulated that the Shc binding site(s) in the PAR_1_ C-tail is/are located upstream of tyrosine 397. Indeed, sequence alignment of PAR_1_ C-tail in nine different species demonstrates several highly conserved regions (data not shown), among which are the Y_381_VY_383_ residues. Replacement of the relevant tyrosine (Y) residues upstream to *Y397Z hPar1* with alanine (Ala, A) (e.g., Y_381_A or Y_383_A and the double mutant Y_381_A & Y_383_A) did not prevent the recruitment and physical association between Shc and PAR_1_ (for more details see section of “Hierarchy of binding”, bellow).

### *Y_381_A-hPar1* exhibits high metastatic potential

**Figure 4:**
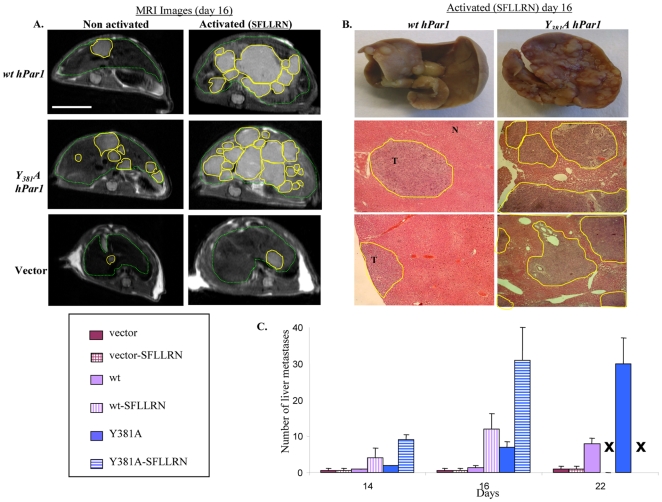
*Y_381_A hPar1* construct enhances liver metastasis formation. **A**. *MRI analysis*. CT-26 mouse carcinoma cells (that do not express endogenous *hPar1*), ectopically forced to express either *wt hPar1* or *Y_381_A hPar1* constructs, were injected intra-splenically into CB6F1 mice to generate liver metastases. Tumor assessment was performed using T_2_W fast SE images (TR/TE = 2000/40 ms). Representative axial liver sections of *wt hPar1* or *Y_381_A hPar1* CT-26-transfected cells, obtained at day 16, in the absence (left panel) or presence (right panel) of SFLLRN, are seen. Liver margins are marked with a dashed green line; yellow lines mark tumor foci; scale bar represents a size of 1 cm and applies to all the images in A. **B**. *Anatomical and histological examination.* Gross anatomical photos (Top) and H&E staining of liver sections (harvested on day 16) of activated *wt hPar1*or *Y_381_A hPar1* CT-26 cells. T = tumor and N = normal; yellow lines mark tumor foci; original magnification ×100. **C.** *Histogram of the number of liver metastases per mouse as measured by MRI.* The experiments were performed in the presence or absence of the PAR_1_ agonist peptide (n = 3–5 mice/group). Activation of PAR_1_ accelerated both tumor size and the number of detectable foci as well as their time of appearance. X stands for sacrificed mice with overloaded liver tumors.

We further demonstrated the functionality of the *Y_381_A hPar1* mutant *in vivo* using a colorectal-liver metastasis model (18), which provides a rapid metastatic model of liver foci formation. Mouse CT-26 colon carcinoma cells were genetically engineered to over-express *wt hPar1*, *Y_381_A hPar1* or empty vector constructs. These over-expressing cells were injected intra-splenically into CB6F1 mice (either PAR_1_ -activated or not) to generate liver metastases. Tumor growth kinetics and liver metastatic foci appearance were monitored twice a week by MRI. Both *wt hPar1* and *Y_381_A hPar1* enhanced liver metastatic foci formation, compared to control CT-26 cells. Furthermore, mice inoculated with cells expressing *Y_381_A hPar1* showed especially extensive and rapid appearance of liver metastasis as compared to mice inoculated with cells over-expressing *wt hPar1* (Fig. 4). Representative MRI images (Fig. 4A) of excised livers and histological sections (Fig. 4B), obtained on day 16, demonstrated high metastatic potential of both activated *Y_381_A hPar1* and *wt hPar1*. An elevated number of metastatic foci were observed with the *wt hPar1* after PAR_1_ activation (Fig. 4A), and an even more dramatic increase was obtained with the activated *Y_381_A hPar1* construct (Fig. 4A, C). Quantification of liver metastasis as a function of time is shown in Figure 4C. These results emphasized that the *Y_381_A hPar1* mutated construct is at least as functional as the *wt hPar1*, and the substitution of Y to A did not impair the ability of *hPar1* to initiate signaling and therefore result in metastasis. The results may further suggest that Shc does not bind directly to PAR_1_ C-tail, since replacement of a key tyrosine residue by alanine (Y381A) does not impair PAR_1_ function as manifested by metastatic foci formation. It is thus postulated that whereas Shc is not associated with PAR_1_ via the traditional tyrosine-phosphorylated-SH2 complex formation, it probably involves a third mediator connecting with PAR_1_. The molecular mechanism of *Y_381_A hPar1*-enhanced liver metastasis remains to be fully elucidated.

### Antibody-array for protein-protein interactions reveals signaling candidates

To detect the putative mediator(s) linking PAR_1_ to potential signaling proteins, we examined custom-made antibody-array membranes. For this purpose, aggressive breast carcinoma MDA-MB-435 cells (with high *hPar1* levels) were incubated with the antibody-array membranes before and after PAR_1_ activation (15 minutes). This identified several activation-dependent proteins which interact with PAR_1_, including ICAM, c-Yes, Shc and Etk/Bmx (see Figure S2). Of these proteins, we chose to focus here on Etk/Bmx and Shc.

**Figure 5:**
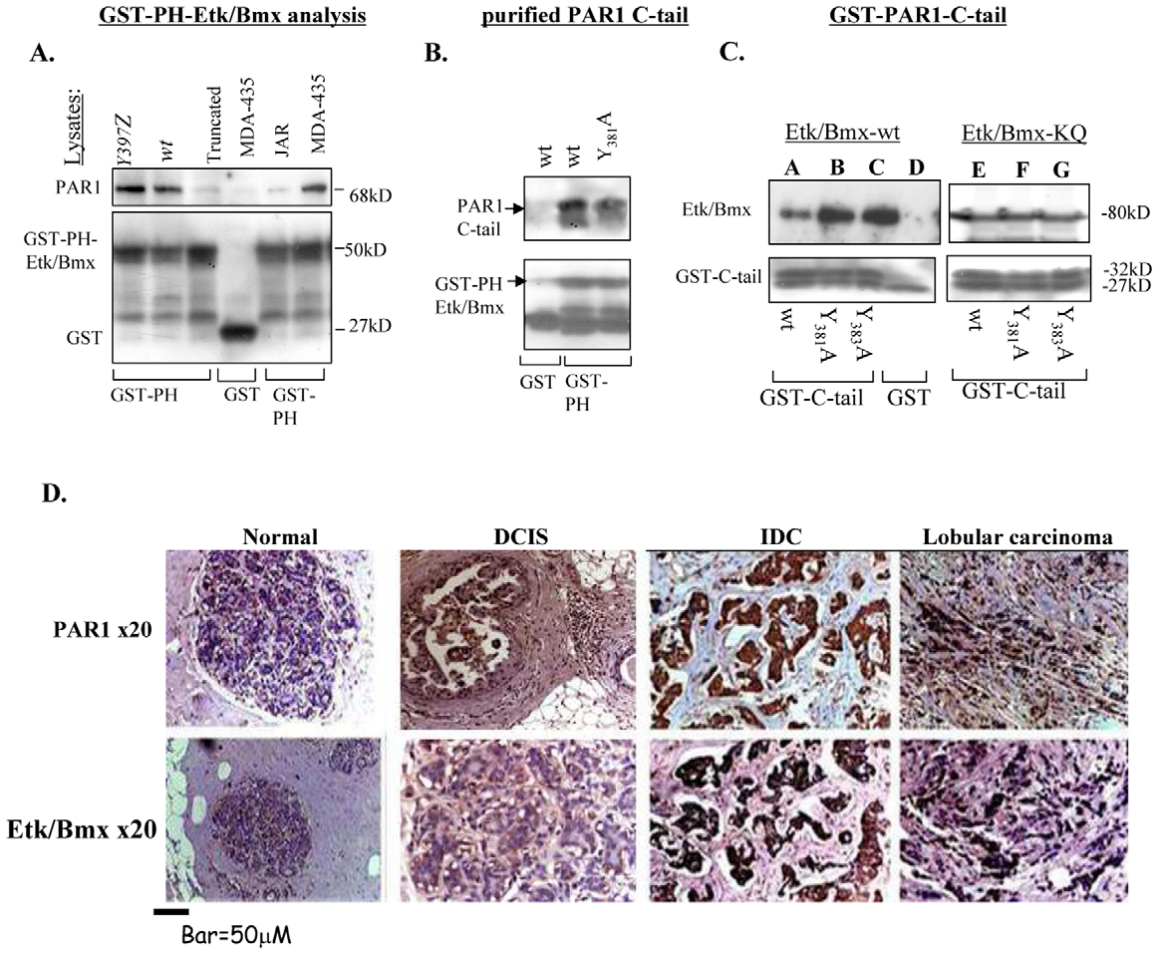
Physical association between PAR_1_ and Etk/Bmx. **A**. *PAR_1_ is physically associated with Etk-PH domain.* Lysates of cells over-expressing *Y397Z*, *wt* or truncated *hPar1*, and lysates of cells that do not express PAR_1_ (e.g., JAR) were applied to a GST-Etk-PH column. Noticeably, highly metastatic MDA-435 cells express high levels of PAR_1_ Specifically-bound proteins were detected using anti- PAR_1_ antibodies. While *Y397Z* and *wt hPar1* showed specific PAR_1_ association, lysates of the truncated *hPar1* or JAR cells showed no binding. **B**. *Purified PAR_1_ C-tail.* PAR_1_ C-tail was cleaved from the immobilized GST-C-tail, purified and re-applied onto a GST-Etk-PH domain column. Specific binding is observed regardless of whether purified *wt* or mutant cleaved C-tail is analyzed. No binding was observed when only GST beads were used. **C**. *GST- PAR_1_ C-tail of wt and mutants.* Lysates of HEK293 cells transfected with either Etk/Bmx (A–D) or kinase-inactive Etk/Bmx (KQ; E–G), applied on various GST-PAR_1_-C-tail columns (PAR_1_-C-tail of *wt*, *Y_381_A*, *Y_383_A*) or GST-control column. The GST-C-tails from either *wt hPar1* or mutants *Y_381_A and Y_383_A* are strongly associated with both *wt* (A–D) and kinase-inactive (KQ; E–G) Etk/Bmx. Control-free GST did not show any binding of KQ Etk/Bmx (data not shown). Specifically-bound proteins were identified using anti-Etk/Bmx antibodies. Levels of GST were used as a control for protein loading. **D**. *Immunohistological staining of PAR_1_ and Etk/Bmx on breast tissue biopsy specimens.* Antibodies directed against PAR_1_ (upper panel) or Etk/Bmx (lower panel) were applied to normal and cancerous breast tissue specimens. The cancerous tissues include DCIS (ductal carcinoma *in situ*), IDC (invasive ductal carcinoma) and lobular invasive carcinoma (lobular carcinoma). The combined histological results were assessed and scored as outlined in the Materials and Methods section. The measurements per slide section was carried out using anatomical compartments, using an ocular micrometer (WHIOX2, Olympus, New Jersey, USA). The microscope was calibrated with a micrometer slide before each measurement. All measurements were performed on the monitor screen using a ×40 objective. On examining the sections for selection of fields tumor cells from the most cellular area at the center of the tumor were selected. Necrotic and inflammatory area were avoided. Eight microscopic fields were screened, 10 cells/field were selected and no less than 50 cells/tumor case were assessed. The positive rate of staining is expressed as a mean ± SD per tumor histological subtype from selected cases. Specific staining is observed in both PAR_1_ and Etk/Bmx, with particularly strong staining seen in IDC and lobular carcinoma. No staining is seen in the normal breast tissue. This staining represents total of 36 cases as outlined for each histological subtype in the table below, performed three times per category.

The epithelial tyrosine kinase (Etk), also known as Bmx, is a non-receptor tyrosine kinase that is unique by virtue of being able to interact with both tyrosine kinase receptors and GPCRs [17]. This type of interaction is mainly attributed to the pleckstrin homology (PH) which is followed by the Src homology SH3 and SH2 domains and a tyrosine kinase site [18]. Etk/Bmx- PAR_1_ interactions were characterized by binding lysates exhibiting various *hPar1* forms to GST-Etk/Bmx. While *Y397Z hPar1* and *wt hPar1* showed specific association with Etk/Bmx, lysates of truncated *hPar1* or JAR cells (lacking PAR_1_) exhibited no binding (Fig. 5A). In order to substantiate the physical association between PAR_1_ C-tail and Etk/Bmx-PH domain we proteolytically cleaved the C-tail portion of both *wt-* and *Y381A hPar1*-modified tail and applied the purified fragments onto a GST-PH Etk/Bmx column. Specific binding was observed with both the *wt hPar1* and *Y_381_A hPar1* purified C-tails (Fig. 5B). Next, we analyzed various modified PAR_1_-GST-C tail constructs (e.g., *wt hPar1, Y_381_A hPar1* and *Y_383_A hPar1*) for binding to either *wt*- or kinase-inactive Etk/Bmx (KQ) cell lysates [18], [19]. A tight association between the PAR_1_ C-tail and Etk/Bmx was obtained, independent of whether *wt hPar1* or the Y/A mutant forms of PAR_1_ C-tail were examined. This was true for both *wt-* and KQ-*Etk* mutant (Fig. 5C).

### Differential expression of Etk/Bmx in breast biopsies

PAR_1_ is highly expressed in breast carcinoma specimens, but not in normal breast tissue, as evidenced by *in situ* hybridization analyses [5], [20]. Immunohistochemical staining of PAR_1_ tissue sections confirms the earlier described RNA riboprobe analysis for *hPar1*. Invasive carcinoma specimens were selected from infiltrating ductal carcinoma (IDC) of high nuclear grade and with evidence of vascular invasion and lymph node metastases. Immunohistological analyses of both PAR_1_ and Etk/Bmx showed little staining in comedo DCIS and ductal carcinoma *in situ*, but high levels of staining in IDC and lobular carcinoma (Fig. 5D; Table 1). These results further suggest a direct correlation between PAR_1_ and Etk/Bmx expression in malignant breast cancer progression. PAR_1_-Etk/Bmx association was also demonstrated by co-immunoprecipitation analysis of MDA-MB-435 cells (expressing both endogenous PAR_1_ and Etk/Bmx) (Figure S3B).

**Table 1:** Expression of PAR_1_ and Etk/Bmx in breast cancer biopsy specimens (representing Fig. 5D).

Histological subtype	Cases(N = 36)	Positive cellsMean ± SD		+1		+2		+3	
		PAR_1_	Etk/Bmx	PAR_1_	Etk/Bmx	PAR_1_	Etk/Bmx	PAR_1_	Etk/Bmx
Normal	12	0.8±0.2	1.2±0.2	0	1	0	0	0	0
DCIS	8	12.5±3.7	14±3.0	2 (25%)	1 (12.5%)	6 (75%)	7 (87.5%)	-	-
IDC	9	40±10.5	42±12.3	2 (22%)	1 (11%)	1 (11%)	0	5 (55%)	6 (66%)
Lobular carcinoma	7	45±7.3	43±11.6	1 (14%)	0	1 (14%)	1 (14%)	6 (85%)	5 (71.4%)

Histological scoring of (N) cases: +1 less than 25% positive cells (weak positive); +2 between 25–75% positive cells (moderate); +3 more than 75% of positive cells (strong). All controls were negative (0–5% positive cells). Extent of expression classified by score (1–3), number of positive cells/field (x = 8).

### Hierarchy of binding

**Figure 6:**
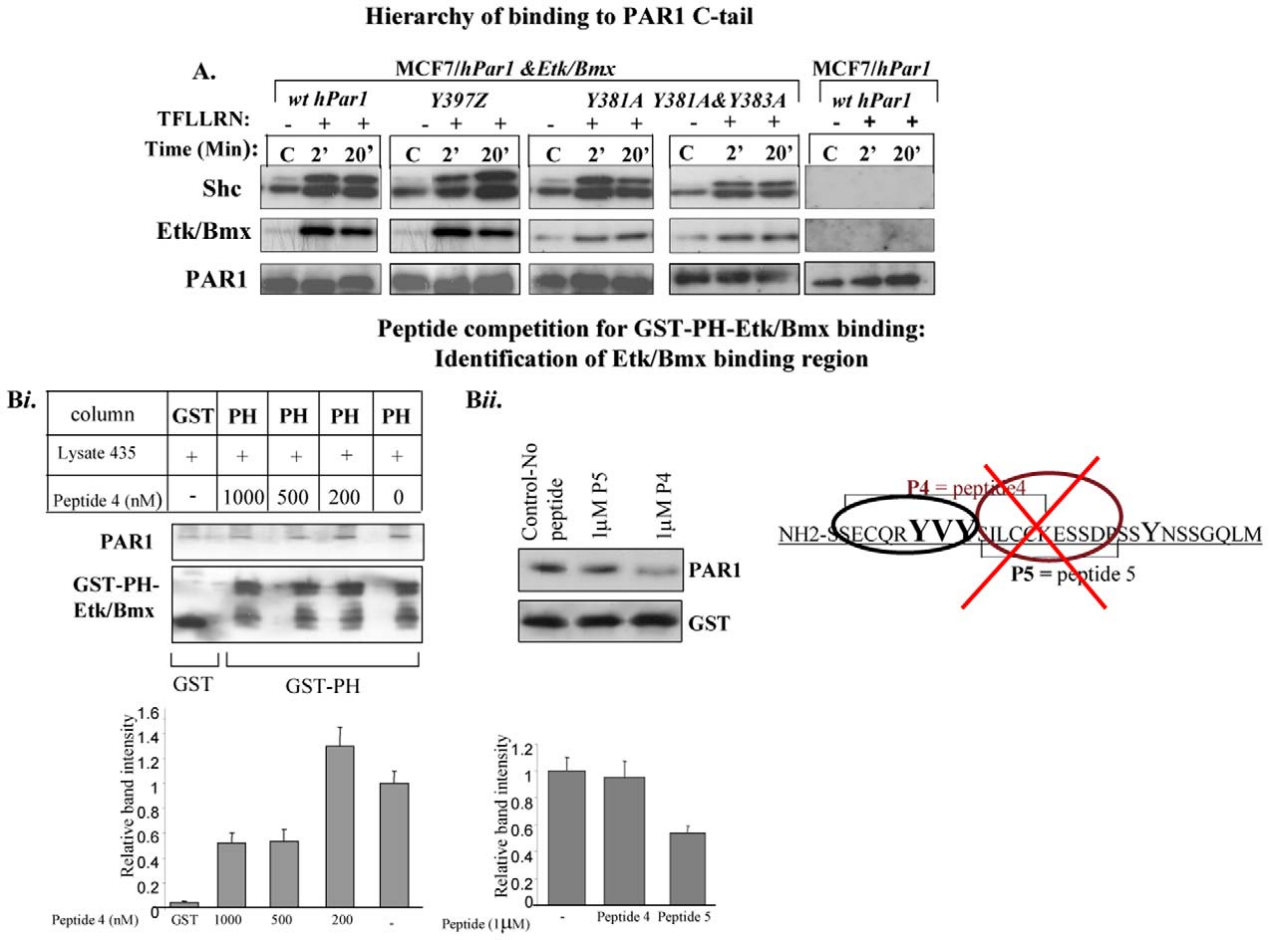
Hierarchy of Shc and Etk/Bmx binding and identification of binding region on PAR_1_ C-tail. **A**. *Etk/Bmx is needed for Shc association with PAR_1_ C-tail.* MCF7 cells before (MCF7/*hPar1*) and after (MCF7/*hPar1*&*Etk/Bmx*) forced Etk/Bmx and *hPar1* co-immunoprecipitation with Shc. No Shc is immunoprecipitated with activated PAR_1_ when Etk/Bmx is absent (right panel: *MCF7/hPar1*; *wt hPar1*). In contrast, extensive co-immunoprecipitation is seen in the presence of forced Etk/Bmx (MCF7/*hPar1*&*Etk/Bmx*). This is true regardless of the various constructs of *hPar1* expressed: *Y_383_A&Y_381_A hPar1*, *Y397Z hPar1*, *Y_381_A hPar1* or *wt hPar1*. The IP was performed using anti- PAR_1_ (ATAP, Santa Cruz, CA). **B**. *Peptide competition for PAR_1_ binding to GST-PH-Etk/Bmx. i.* Peptide 4, encompassing residues 375–396 of PAR_1_ C-tail, competes with PAR_1_ for binding to the GST-PH-Etk/Bmx. Specific binding of PAR_1_to the GST-PH-Etk/Bmx is inhibited in the presence of increased peptide concentration (200 nM-1 µM). *Lower panel*: Representative histograms show the relative intensities of the bands expressed as a ratio of PAR_1_to GST-PH. **B** *ii*. Peptide 5, representing another region (e.g., 379–402), does not compete at a concentration of 1 µM. GST protein serves as a loading control. *Lower panel*: Representative histograms show the relative intensities of the bands expressed as a ratio of PAR_1_ to GST.

Next, we wished to determine the chain of events mediating the signaling of PAR_1_ C-tail-Shc and Etk/Bmx. To this end, analysis of MCF7 cells that express little to no *hPar1* were ectopically forced to over-express *hPar1*. When co-immunoprecipitation with anti- PAR_1_ antibodies following PAR_1_ activation was performed, surprisingly, no Shc was detected in the PAR_1_ immunocomplex (Fig. 6A; MCF7/*hPar1*; right panel). Shc association with PAR_1_ was fully rescued when MCF7 cells were initially co-transfected with Etk/Bmx (Fig. 6A), with abundant assembly of Shc in the immunocomplex. Thus, Etk/Bmx is a critical component that binds to activated PAR_1_ C-tail and enables subsequent binding of Shc. Shc may bind either to phosphorylated Etk/Bmx, via the SH2 domain, or in an unknown manner to the PAR_1_ C-tail, provided that Etk/Bmx is present and is PAR_1_ -bound. One cannot, however, exclude the possibility that Bmx binds first to Shc and only then does the complex of Etk/Bmx-Shc bind to PAR-1.

### Identification of PAR_1_-Etk/Bmx binding region: functional consequences

Peptides (representing various regions in PAR_1_ C-tail) were used in a competition analysis assay for the binding of PAR_1_ cell lysates to GST-PH-Etk/Bmx. An 18-amino-acid peptide encompassing residues 375–392 of PAR_1_ C-tail (e.g., termed peptide 4) yielded a dose-dependent inhibition within the range of 1–1000 nM applied peptide (Fig. 6B). Two other peptides, representing PAR_1_ C-tail 387–400 (Fig. 6B *ii*; termed peptide 5) or residues 393–412 (data not shown), did not compete.

**Figure 7:**
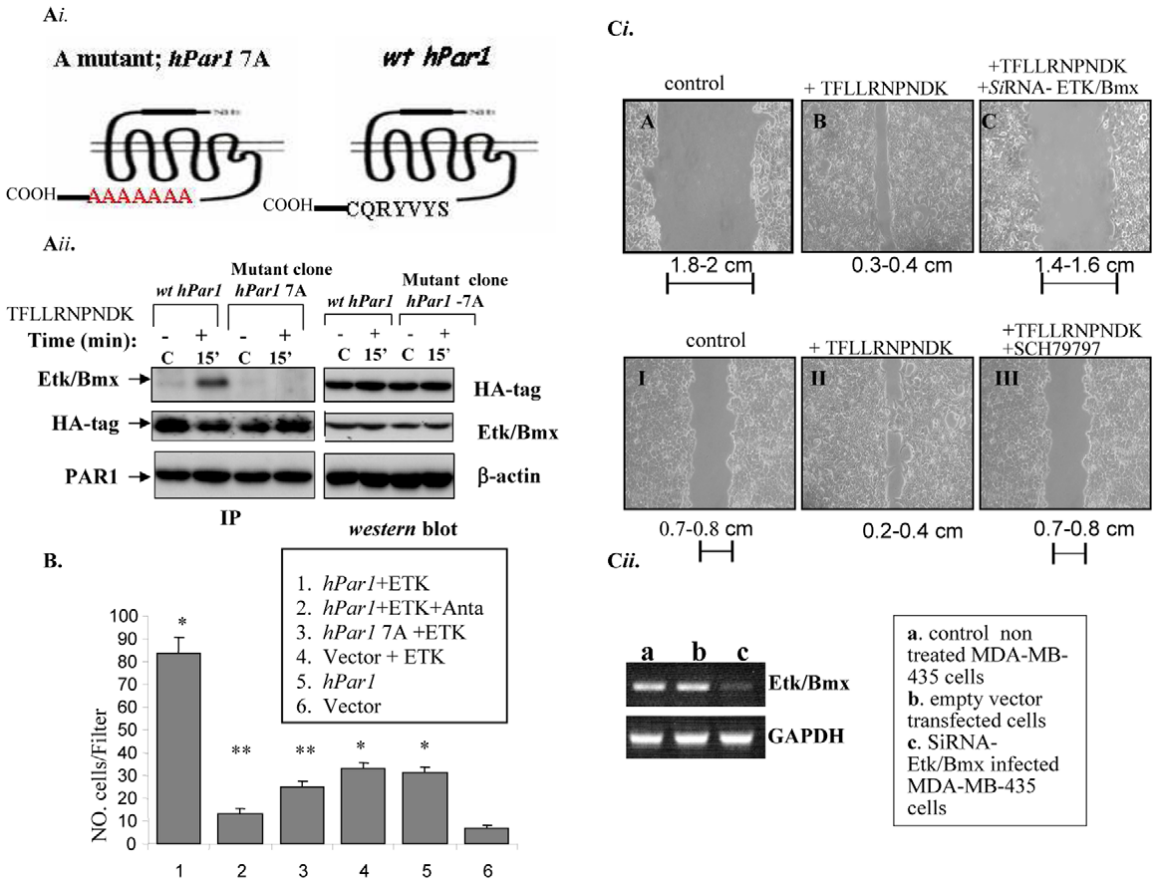
Functional consequences of mutant *hPar1-7A* versus *wt hPar1*. **A** *i*. Schematic representation of *wt hPar1* and the mutant *hPar1-7A*. **A** *ii*. *The ectopic expression of hPar1-7A mutant construct abrogates binding of Etk/Bmx to PAR_1_ C-tail.* Stable clones of either HA-tagged *wt hPar1* and/or a mutant construct of HA-*hPar1-*7A were activated and further analyzed for co-immunoprecipitation of PAR_1_ with Etk/Bmx. The IP was carried out using anti-HA (sc-7392; Santa Cruz, CA). The blots were detected by anti-Bmx (Transduction Laboratories, BD Biosciences, CA) for the identification of Etk/Bmx-associated PAR_1_. Levels of the HA-tag (for PAR_1_) are shown in the middle panel. Similarly, levels of PAR_1_ are also shown by application of anti- PAR_1_ (ATAP; Santa Cruz, CA) (lower panel). The right section shows levels of plasmid transfection efficiencies in the cells, as indicated by HA- PAR_1_ and Etk/Bmx analysis by western blots. As seen, only the *wt hPar1* co-immunoprecipitated with Etk/Bmx (*wt hPar1*), while the mutant HA-*hPar1-*7A clone (mutant clone *hPar1- 7A*) failed to co-immunoprecipitate. This takes place under conditions whereby both constructs are well expressed, as evidenced by anti-HA-antibodies (western blot; right panel). **B**. *Matrigel invasion assay of either MCF7 cells stably expressing HA-wt-hPar1and Etk/Bmx, or HA-hPar1-7A mutant and Etk/Bmx.* Invading cells were counted and the mean ± SD of ten fields per filter was determined. Stable HA *wt hPar1* clone or HA-*hPar1-*7A mutant clone were co-transfected with Etk/Bmx construct and TFLLRNPNDK-activated. Marked Matrigel invasion is seen in the *wt hPar1* and Etk/Bmx clones. Low invasion levels are observed in the presence of *hPar1-7A* mutant and Etk/Bmx, *hPar1* alone, empty vector, vector and Etk/Bmx or PAR_1_ antagonist. These data are representative of three experiments. **C** *i*. *Migration assay for wounding-scratch monolayer cells.* MDA-MB-435 cell monolayer (expressing endogenous Etk/Bmx) were scratched to introduce an equal gap-area under the following conditions: control untreated cells, TFLLRNPNDK-activated or *Si*RNA-Etk/Bmx and TFLLRNPNDK-activated. In this set, rapid closure of the wound was obtained 24 hours under PAR_1_ activation as compared to control untreated cells. In contrast, no migration and closure of the wound was seen when the Etk/Bmx level of the cells were knocked down and they were TFLLRNPNDK-activated. In another set of wound introduction experiments, attenuated wound closure was seen in the presence of the V antagonist SCH79797 and SFLLR NPNDK PAR_1_ activation for 24 h, similar to the control untreated cells. These data are representative of four experiments. **C** *ii*. RT-PCR analysis showing the level of Etk/Bmx in MDA-MB-435 cells before and after SiRNA- Etk/Bmx cell infection.

Based on the competition assay we prepared mutated *hPar1* constructs with successive replacement of seven residues (378–384; CQRYVYS). MCF7 clones expressing either HA-*hPar1*-7A or HA-*wt hPar1 s*howed the following outcome (characterization and proper expression of the mutant is shown in Figure S4): HA-*wt* following activation (Fig. 7A). We thus conclude that the critical region for Etk/Bmx binding to PAR_1_ C-tail resides in the vicinity of CQRYVYS.

Activated MCF7 *hPar1*-7A mutant cells (also expressing Etk/Bmx) failed to invade Matrigel-coated membranes, as compared to a potent invasion level obtained by activated *wt hPar1* (Fig. 7B).

**Figure 8:**
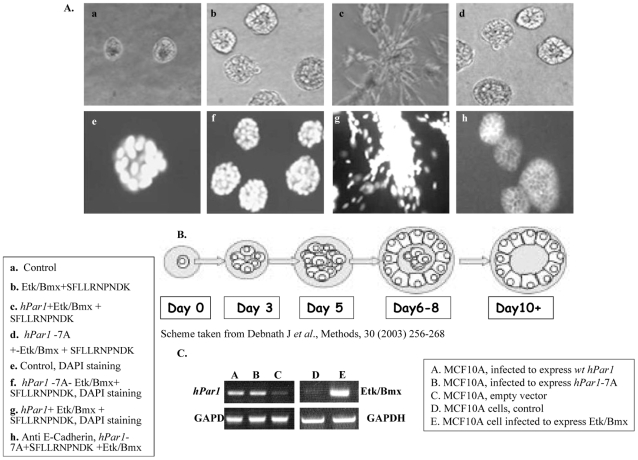
Morphogenesis of MCF10A spheroids infected with either *wt hPar1*, mutant *hPar1-7A* or with Etk/Bmx maintained in 3-D Matrigel cultures. **A**. **Upper panel.** Representative phase-contrast microscopic images of MCF10A cells under the following conditions: a. control untreated MCF10A. b. MCF10A cells infected with Etk/Bmx and SFLLRNPNDK PAR_1_ -activated. c. MCF10A cells infected with both *wt hPar1* and Etk/Bmx and SFLLRNPNDK-activated. d. MCF10A cells infected with both mutant *hPar1-7A* and Etk/Bmx and SFLLRNPNDK-activated. **Lower panel.** Representative confocal microscopic images of MCF10A acini. e. Control untreated MCF10A, DAPI stained nuclei in a representative spheroid. f. MCF10A cells infected with both the mutant *hPar1-7A* and Etk/Bmx and SFLLRNPNDK-activated; DAPI staining of spheroid nuclei. g. MCF10A cells infected with both the *wt hPar1* and Etk/Bmx and SFLLRNPNDK-activated; DAPI staining of the nuclei. h. MCF10A cells infected with both the mutant *hPar1-7A* and Etk/Bmx and SFLLRNPNDK-activated, stained for cell-cell contact with anti-E-cadherin. **B**. Schematic representation of MCF10A spheroid development in 3-D Matrigel cultures. **C**. RT-PCR analyses for the different MCF10A-infected cells. Levels of expression as compared to those of a house-keeping gene (GAPDH). A. MCF10A-infected *wt hPar1* B. MCF10A infected with mutant *hPar1*-*7A*. C. Empty vector infected-MCF10A cells. D. Control, untreated MCF10A cells. E. MCF10A cells infected with Etk/Bmx.

In a parallel experiment, a wound assay for the rate of cell migration, showing the ability of the cells to fill in gaps in an MDA-MB-435 cell monolayer, was performed. Rapid closure of the *hPar1*, but not HA-*hPar1*-7A, exhibited binding association with Etk/Bmx wound was observed following TFLLRNPNDK PAR_1_ activation. This PAR_1_ -induced cell migration was markedly inhibited when the cells were infected with siRNA-Etk/Bmx construct to knock down the endogenous Etk/Bmx levels present in MDA-MB-435 cells (Fig. 7C, top panel). Similar inhibition was obtained in the presence of the PAR_1_ antagonist, SCH 79797 (Fig. 7C, bottom panel), pointing to the important role of both PAR_1_ and Etk/Bmx in wound closure/migration of PAR_1_-activated MDA-MB-435 cells. The highly ordered tissue organization of normal epithelia is aggressively disrupted in pathological conditions. This is well recapitulated in the MCF10A cell-growth model, mimicking epithelia apico-basal polarity (24). We examined the morphogenesis of MCF10A mammary acini in three-dimensional (3-D) basement membrane cultures. Normal-appearing intact spheroids are formed in the presence of control Etk/Bmx-expressing MCF10A cells and activation by SFLLRNPNDK, a PAR_1_ agonist peptide (Fig. 8A; a, b). In contrast, in the presence of ectopically forced *hPar1* (expressing also Etk/Bmx) and following PAR_1_ activation, an oncogene-like, migratory morphogenesis was obtained which was characterized by a complete loss of the cell-cell tight junction contacts and the invaded basement membrane architecture (Fig. 8A; c and g). Significantly, when the MCF10A cells (in the presence of endogenously expressed Etk/Bmx) were infected with the mutant cytoplasmic form of *hPar1* (*hPar1-*7A) and SFLLRNPNDK PAR_1_ -activated, nearly normal-appearing spheroid morphology was obtained (Fig. 8A; d, f). This outcome highlights the fact that by preventing immobilization of Etk/Bmx on PAR_1_ C-tail, inhibition of invasion and lack of apico-basal polarity morphogenesis of an oncogene-like phenotype in MCF10A cells are observed.

## Discussion

In the present study we characterized the contribution of PAR_1_ signaling events in breast cancer progression. We utilized constructs of *hPar1*, including *wt hPar1* and either deleted or mutant *hPar1* forms, and analyzed them for their ability to induce tumor development in mouse mammary gland and colorectal-liver metastasis models *in vivo*, as well as characterizing them biochemically *in vitro*. Injection of cells expressing *wt hPar1* or *Y397Z hPar1* into mouse mammary glands resulted in pronounced mammary tumor growth and angiogenesis. The truncated PAR_1_ construct, however, which lacked the cytoplasmic tail, was incapable of promoting PAR_1_ -induced tumors *in vivo*. These findings emphasize the pivotal role of the cytoplasmic tail in PAR_1_ function. Antibody-array analyses for the detection of signaling partners that bind to PAR_1_ revealed the involvement of Shc and Etk/Bmx, among others. We describe herein a novel signaling complex formed between Etk/Bmx and Shc assembled onto the PAR_1_ C-tail, thus providing a central docking site to facilitate tumor progression, invasion and angiogenesis.

Studies of *Par1*-deficient mice revealed a critical role for PAR_1_ in blood vessel formation [13]. PAR_1_ induces tumor angiogenesis via the up-regulation of at least VEGF and Gro oncogenes (27,28). Furthermore, *hPar1* gene withdrawal leads to the selective regression of immature but not mature blood vessels [21]. We show here that tumors generated by either *wt hPar1* or persistent *Y397Z hPar1* are capable of inducing marked vascularization, as compared to only a few blood vessels formed by the truncated *hPar1*-induced tumors. Together, these results emphasize a central role for PAR_1_ expression and signaling in tumor progression.

A critical role for Etk/Bmx and Shc was demonstrated by their selective recruitment to activated PAR_1_ C-tail. Moreover, the prime event of Etk/Bmx binding is a prerequisite for further PAR_1_-Shc association. We demonstrate herewith the hierarchy and sequence of events during PAR_1_ signaling in breast cancer progression. While PAR_1_-induced Shc phosphorylation has been previously reported [14], [22], [23], we provide here the first evidence for a physical association between Shc and PAR_1_. Substitution of tyrosine residues in PAR_1_ C-tail by alanine did not abrogate the recruitment of Shc to PAR_1_ tail, indicating that PAR_1_ tyrosine residues are not involved in the direct association between PAR_1_ and Shc. Furthermore, the mutant Y_381_A showed enhanced metastatic capabilities when compared to *wt hPar1* in a colorectal-liver metastasis animal model. This implies that the Y/A substitution (Y_381_A) most likely endows PAR_1_ with better accessibility to essential signaling proteins and thereby enables enhanced metastasis (a hypothesis that is currently under investigation). The fact that the tyrosine residues in PAR_1_ C-tail do not play a role in the binding of Shc implies the requirement of a third mediating partner. Indeed, Etk/Bmx binding to PAR_1_ C-tail through the PH-domain is required for PAR_1_ -Shc association. One cannot, however, exclude the possibility that Bmx binds first to Shc and only then does the complex of Etk/Bmx-Shc bind to PAR_1_.

The fact that we have obtained tumors *in vivo* following implantation of either MCF7 cells over-expressing *wt hPar1, or Y397Z hPar1* in the mammary fat pads of mice (Fig. 1), raises the issue as to the significance of Etk/Bmx *in vivo*. When we have immunostained for Etk/Bmx, xenograft sections derived from the tumors obtained of MCF7 cells over-expressing these constructs, abundant and high levels of Etk/Bmx was found in tumors generated by MCF7/*wt hPar1* and MCF7/*Y397Z hPar1*. In-contrast, no Etk/Bmx was seen in either MCF7/empty vector or MCF7/truncated *hPar1* small tumors (see Figure S5). This finding recapitulates the presence of Etk/Bmx in tumors generated by MCF7 cells over-expressing *hPar1, in vivo* in a manner that remains to be fully explored. One attractive possibility is that environmental cues in-vivo are responsible for the expression of Etk/Bmx in PAR1-derived tumors. It is plausible that the very low endogenous levels of Etk/Bmx originally present in MCF7 cells are markedly induced *in vivo*, due to micro environmental signals (in a yet unknown manner).

The recruitment of Etk/Bmx and Shc to PAR_1_ C-tail provides a bridge linking PAR_1_ with receptor tyrosine kinases (RTKs) and associated downstream signaling pathways. For example, the proto-oncogene Src plays a pivotal role in integrin activation during tumor progression [24]. While the C-terminal Src kinase consists of SH3, SH2 and kinase domains, it may be recruited to a phosphorylated site in Etk/Bmx (following PAR_1_ activation; see Fig. 3A) via the SH2 domain. This scaffold assembly of PAR_1_ -Etk/Bmx-Shc and Src complex formation remains to be fully elucidated.

Whereas Etk has been shown to mediate integrin signaling by binding to the FAK FERM domain through the Etk PH domain [25], activation of PAR_1_ has also been shown to induce FAK phosphorylation and integrin signaling via cross talk with αvβ5 integrin [26]. However, it is not known how PAR_1_ activation leads to α_v_β_5_ integrin activation and FAK phosphorylation. While, in breast cancer, activated PAR_1_ is engaged in binding Etk/Bmx via the PH domain, thus occupying this site, it is plausible that the Pro-712/713 proline-rich region of FAK (e.g., Pro-X-X-Pro-X-Arg) associates with the SH3 domain present in Etk/Bmx, connecting PAR_1_ to integrins via FAK association to Etk/Bmx. This possibility needs to be fully explored. It has also been demonstrated that the docking protein p130Cas forms a complex with Etk/Bmx. This protein plays a role in the formation of focal adhesion complex sites, the regulation of actin cytoskeleton reorganization and cell migration. Since Cas undergoes tyrosine phosphorylation prior to complex formation, it is feasible that phosphorylated p130Cas associates with the SH2 domain of PAR_1_ -C-tail immobilized Etk/Bmx [27].

PAR_1_ induces activation of the small GTPase RhoA, which participates in formation of focal adhesions and in regulating the reorganization of the actin cytoskeleton. It was suggested that Etk/Bmx activates Rho A by releasing the GDI from the inactive RhoA-GDI complex through the interaction with Etk/Bmx PH domain [28]. Perhaps a balance is obtained between the free Etk/Bmx-PH domain in activating RhoA and the PAR_1_-C-tail immobilized Etk/Bmx-PH domain, in the delicate control of cytokeletal reorganization and actin stress fiber formation during breast cancer progression.

Akt/PKB, first identified as the cellular homologue of the transforming oncogene *v-Akt* [29], is a core component of the phosphoinositide 3-kinase (PI3K) signaling pathway and effectively promotes cancer cell survival and proliferation. Activation of PI3K may be mediated via association of the SH2 domain in one of the two (85 kDa and 110 kDa) PI3K domains (most likely p85) with phosphorylated tyrosine residues in Etk/Bmx, following PAR_1_ activation. This leads to the recruitment of PI3K to the cell membrane and ultimately to activation of the serine/threonine kinase Akt, an immediate downstream target acting to promote breast cancer survival.

Shc in the complex (PAR_1_ -Etk/Bmx-Shc) may recruit the Grb2 (growth factor-bound protein-2)-SOS (Son of sevenless) complex to the membrane via binding of the SH2 domain of Grb2 to the phosphotyrosine on Shc. SOS, a GEF for Ras, can then exchange the GDP bound to Ras GTP. Once Ras binds GTP, it is then activated, leading to the ERK activation cascade.

Increasing evidence suggests that the *Btk* kinase family plays a central role in various cellular processes [18]. Recently, *Bmx* transgenic mice were demonstrated to exhibit skin hyperplasia, inflammatory cell recruitment and strong dermal angiogenesis, as well as accelerated wound healing [30]. In agreement with these transgenic animal characteristics, Etk/Bmx was shown to interact physically with a vast number of proteins, among them FAK [25], *Src* [31], G-proteins [32], [33], [34], STAT3[19] and P21[35]. We found that Etk/Bmx kinase is phosphorylated following PAR_1_ activation (see Fig. 3A), suggesting that kinase activation is important for the downstream signaling of PAR_1_. A seven-amino-acid sequence region in the PAR_1_ C-tail portion was identified as the site required for association with the Etk/Bmx-PH domain. Part of this region, located in the 8^th^ helix region, as revealed by the X-ray structure of rhodopsin, was previously reported to be significant for platelet PAR_1_ activation [36].

PAR_1_ C-tail is a highly conserved region among species, implying the importance of this region in *protein-protein* interaction and signaling. Stable MCF7 clones expressing both *wt hPar1* and Etk/Bmx exhibit enhanced invasion properties potently inhibited by a PAR_1_ -specific antagonist. In contrast, low levels of invasion are shown by stable MCF7 clones of Etk/Bmx and *hPar1-*7A mutants. Similar data, further supporting the importance of Etk/Bmx in PAR_1_ -induced migration, is demonstrated by wound-gap closure in cell monolayers. The key role of Etk/Bmx is also shown in the nearly normal acinar structure of MCF10A cell cultures maintained in a three-dimensional (3-D) environment *in vitro*. This 3-D cell culture mimics large, epithelial tissues *in vivo*, in the context of apico-basal polarized structure. The ectopic forcing of *wt hPar1* in MCF10A cells, followed by activation, results in dramatic altered structures of disrupted tight-junction contacts, complete loss of cell polarity and extensive protrusions of invaded basement membranes. Blocking Etk/Bmx binding to PAR_1_ by over-expressing *hPar1*-7A mutant in MCF10A cells maintained in 3-D basement membrane cultures abrogates the oncogene-like phenotype. Instead, nearly ball-shaped spheroids of apico-basal polarization are obtained. This outcome strongly supports a fundamental role for Etk/Bmx in PAR_1_ -induced invasion. Our data contribute to a list of Etk/Bmx counter-partners, such as TNF, that mediate angiogenesis via binding to a 16-amino-acid sequence of TNFR2, which binds to multiple Etk/Bmx binding domains (PH, TEC and SH2) [37]. Similarly, the Etk-PH-domain has been shown to regulate integrin signaling, and promote cell migration through the specific association of FAK via the FERM domain [25]. In addition, Etk/Bmx immobilized to PAR_1_ C-tail may provide a central junction site for possible cross-talk between PAR_1_ and EGFR, as well as ErbB2/HER2, following PAR_1_ activation [38].

Our study identifies essential signaling partners in PAR_1_ -mediated breast cancer progression, determines the hierarchy of binding and identifies a critical associating region in the PAR_1_ C-tail. This is the first demonstration of the PAR_1_ C-terminus serving as a scaffold tail. The identification of a PAR_1_ C-tail binding domain may provide a platform for new therapeutic vehicles in the treatment of breast cancer.

## Materials and Methods

### Cell culture

MCF7 and MDA-MB-435 human breast carcinoma, CT-26 mouse colon carcinoma, HEK-293 cells and the African green monkey kidney fibroblast cell line COS1 (these cell lines were obtained from the ATCC, VA, USA) were maintained in DMEM with 10% fetal calf serum. Stable clonal cell lines over-expressing *wt hPar1*, *Y397Z hPar1*, truncated *hPar1* and Y/A mutants; *Y_381_A&_383_A hPar1*or the *wt*-Etk and Etk-KQ were selected for G418 resistance (800 µg/ml).

### Plasmids and transfection

MCF7 cells were transfected with 1–2 µg of either *wt* human *hPar1* or truncated *hPar1* or *Y397Z hPar1* cDNA, or with a control pcDNA3 vector (Invitrogen, Carlsbad, CA) using FuGene transfection reagent (Roche Molecular Biochemicals, Indianapolis, IN). Transfected cells were selected with G418 (800 µg/ml) to obtain stable populations of cells expressing *hPar1* and the variants. Etk/Bmx plasmids (e.g., *wt*, kinase-dead, KQ and GST-PH-Etk/Bmx) [18], [19] were transfected into HEK-293 cells using the same protocol as previously described.

### RNA isolation and RT-PCR

RNA was isolated with Tri-Reagent (MRC, Cincinnati, OH) according to the manufacturer's instructions. After reverse transcription of 1 µg total RNA by oligo (dT) priming, cDNA was amplified using Taq DNA polymerase (Promega, Madison, WI). Comparative semi-quantitative PCR was performed using the following primers: GAPDH sense: 5'-CCA CCC ATG GCA AAT TCC ATG GC-3' and antisense: 5'-TCT AGA CGG CAG GTC AGG TCC ACC-3' primers. PAR_1_ N-terminus primers were as follows: *hPar1*-sense: 5′- CTCGTCCTCAAGGAGCAAAC-3′, antisense orientation: 5′-TGGGATCGGAACTTTCTTTG-3'.

(resulting with a 564-bp PCR product). PAR_1_ C-tail primers - sense: 5′-TAC TAT TAC GCT GGA TCC TCT GAG-3′ and antisense: 5′-CTT GAA TTC CTA AGT TAA CAGCTT-3′. These primers give rise to a 181-bp product corresponding to the entire PAR_1_ C-tail site, as follows:

YY - YASSECQRYVYSILCCKESSDPSYNSSGQLMASKMDTCSSNLNNSIYKKLLT.

### Animal studies: Mammary gland model

Female athymic nude mice at 6–8 weeks of age were pre-implanted subcutaneously with pellets containing 1.7 mg β-estradiol (60-day release, Innovative Research of America, Sarasota, FL). Mouse mammary pads were then injected with 1×10^7^ MCF-7 cells stably transfected with *hPar1 wt* and mutant constructs (e.g., *wt*, *Y397Z* and truncated) or pcDNA3 control plasmid. Mice were monitored for tumor size by external caliber measurements (length and width) on days 10, 22, 25, 29, 33, 36 and 45. Tumor volume (V) was calculated by V = L×W2×0.5, where L is length and W is width. On day 45, mice were sacrificed and tumors were removed, weighed and fixed in formalin for histology. All animal experiments were approved by the animal committee of the Hebrew University, Jerusalem, Israel (MD-107.05-4).

### Liver metastasis model

CT-26 mouse colon carcinoma cells were stably transfected with either *wt hPar1* or *hPar1-Y_381_A* constructs. The activation of PAR_1_ (using the peptide SFLLRN) was performed prior to injection into the mice. CB6F1 mice were anesthetized (75 mg/kg ketamine +3 mg/kg xylazine, i.p.), and the spleen was exteriorized through an incision (1.0 cm) on the left side of the mouse. CT-26 cells (10^4^ cells/mouse) transfected with the different constructs (e.g., *hPar1*, *hPar1 Y381A*, mock-transfected vector) were injected into the spleen using a 30-gauge needle. The cell suspension was allowed to enter the portal circulation over a short period (5 minutes), after which the spleen was removed, as previously described [39]. The wound was sutured and the animal was allowed to recover. MRI images were monitored every 2–3 days on a 4.7T Bruker Biospec spectrometer using a bird-cage coil. Tumor assessment was made by serial coronal and axial T_2_W fast SE images (TR/TE = 2000/40 ms). All experiments were performed in accordance with the guidelines of the Animal Care and Use Committee of the Hebrew University, Jerusalem, Israel (MD-107.05-4).

### PAR_1_ activation

PAR_1_ was activated by the SFLLRN (H-Ser-Phe-Leu-Leu-Arg-Asn-NH_2_) peptide, the TFLLRNPNDK peptide, a selective PAR_1_ agonist, or thrombin (1 U/ml).

### Histology

Tissue samples derived from the primary tumors were fixed with 4% formaldehyde in PBS, embedded in paraffin and sectioned (5-µm sections). After de-paraffinization and re hydration, sections were stained with hematoxylin and eosin (H&E) or subjected to immunohistochemistry using specific antibodies.

### Histological evaluation and scoring

The combined histological results were assessed and scored as previously described[40], [41]. The measurements per slide section was carried out using anatomical compartments, using an ocular micrometer (WHIOX2, Olympus, New Jersey, USA). Slides review was independently performed by two investigators (BM and RB). Discrepancies were resolved by simultaneous re-examination of the slides by both investigators using a double-headed microscope. The microscope was calibrated with a micrometer slide before each measurement. All measurements were performed on the monitor screen using a ×40 objective. On examining the sections for selection of fields tumor cells from the most cellular area at the center of the tumor were selected. Necrotic and inflammatory area were avoided. Eight microscopic fields were screened, 10 cells/field were selected and no less than 50 cells/tumor case were assessed. The positive rate of staining is expressed as a mean ± SD per tumor histological subtype from selected cases.

### Immunohistochemistry

Sections were subjected to inactivation of endogenous peroxidase (3% H_2_O_2_ in DDW), antigen retrieval by microwave oven (3 min) in citrate buffer (0.01 M, pH 6.0), and blocking with 10% goat serum in PBS. Sections were then incubated with antibodies directed against Von-Willebrand factor (anti-factor VIII, DAKO, Carpinteria, CA), Ki-67 (Clone SP6, Lab Vision-NeoMarkers, Fremont, CA), or an endothelial cell-specific lectin (*Bandeiraea simplicifolia* BS-1 isolation) [42], followed by incubation with horseradish peroxidase-conjugated anti-rabbit antibody (DAKO, Carpinteria, CA). Color was developed by incubation (10 min) with the Zymed AEC substrate kit (Zymed Laboratories, South San Francisco, CA), and counterstained with Mayer's hematoxylin.

### Preparation of *hPar1* constructs: truncated *hPar1*, *Y397Z hPar1*, *Y_381_A hPar1* and *Y_383_A hPar1*

Detection of *hPar1* was carried out using primers: sense orientation: 5′- CTCGTCCTCAAGGAGCAAAC-3′, antisense orientation: 5′-TGGGATCGGAACTTTCTTTG-3'. For the PAR_1_ -C-tail primers: sense orientation: 5′ -TACTATTACGCTGGATCCTCTGAG-3′, antisense: 5′- CTTGAATTCCTAAGTTAACAGCTT-3′. These primers give rise to a 181-bp product corresponding to the entire PAR_1_ C-tail site, as follows:

YY - YASSECQRYVYSILCCKESSDPSYNSSGQLMASKMDTCSSNLNNSIYKKLLT

Using polymerase chain reaction, we constructed a PAR-1 mutant protein truncated in its cytoplasmic tail after amino acid leucine 369 or at tyrosine 397. Truncated *hPar1* at residue 369 was designed as described in [14]. *Y397Z* construct: PAR-1 cDNA served as a template for amplifying the fragment containing STOP codon using the followed primers: sense: 5'-ATA AGC ATT GAC CGG TTT CTG-3' and antisense: 5'-GCT CTA GAT TTT AAC TGC TGG GAT CGG AAC-3'. Replacement of tyrosine residues at PAR_1_ cytoplasmic tail was achieved using specific primers containing the point mutation. Primer sequences were as follows: 381-sense: 5'-TGC CAG AGG GCT GTC TAC AGT ATC TTA TGC-3', 381-antisense: 5'- GAT ACT GTA GAC AGC CCT CTG GCA CTC AGA-3', 383-sense: 5'-GCC AGA GGT ACG TCG CAA GTA TCT TAT GCT GCA AA-3', 383-antisense: 5'-AAG ATA CTT GCG ACG TAC CTC TGG CAC TCA G-3'. The amplified DNA fragment was digested with *Xba*I and *Hind*III from PAR_1_ cDNA and cloned into a pcDNA3 plasmid, followed by DNA sequencing. To confirm the functional integrity of the DNA constructs, *wt* and mutant cDNAs were transiently expressed in COS-1 cells that were subsequently subjected to FACS analysis with a PAR-1-specific antibody (WEDE15-PE, Immunotech, Cedex, France).

### HA-tag *wt hPar1* and HA-mutant *hPar1-*7A C-tail constructs

The mutants were designed for insertion of A at the carboxy terminus of PAR_1_ residues 378–384: SSE**CQRYVYS**ILCCKESS to SSE**AAAAAAA**ILCC (named *hPar1*-7A mutant). For HA-tag *wt hPar1* construct PCR primers were designed and added downstream to the ATG start codon. Primers for the HA-tag are as follows: sense: 5'- TAC CCA TAC GAT GTT CCA GAT TAC GCT-3' and anti-sense: 5′-AGC GTA ATC TGG AAC ATC TA TGG GTA-3′. Replacement of seven residues with Ala (A) at positions 378–384 was made by synthesis of oligos containing the mutation. Primer sequences were as follows: *hPar1* 7A mutant: sense: 5'- TCT GAG **GCT GCT GCT GCT GCT GCA GCT** ATC TTA -3' and anti-sens:e 5'- TAA GAT AGC TGC AGC AGC AGC AGC AGC CTC AGA -3'. PCR products were then used as primers on an *hPar1* cDNA template to create an extended product of introduced mutations into the full-length sequence. The amplified DNA fragment was digested with *Pin*AI and *Xba*I from PAR_1_ cDNA and cloned into pcDNA3-*hPar1* plasmid followed by DNA sequencing.

### GST-C-tail cloning

GST-C-tail of PAR_1_ fragment, containing 54 amino acids from serine 369 to residue 425, was prepared using RT-PCR (5′-TAC TAT TAC GCT GGA TCC TCT GAG-3′ and 5′-CTG AAT TCC TAA GTT AAC AGC TT-3′). The resulting DNA fragment was further cut with the appropriate restriction enzymes (*Bam*H1 and *Eco*R1) and ligated into pGEX2T vector. The GST-C-tail was separated by SDS-PAGE, which indicated that the fusion protein of the C-tail was adequately prepared. The molecular weight of GST protein is 27 kD and the GST-C tail fusion protein is 32 kD. GST-Shc-SH2 and tandem SH2 were kindly provided by S. Katzav, Hubert H. Humphrey Center for Experimental Medicine and Cancer Research, Hebrew University-Hadassah Medical School, Jerusalem.

### GST fusion protein columns

Fusion proteins were purified from transformed *Escherichia coli* bacteria that had been stimulated with isopropyl-β-D-thio-galactopyranoside (IPTG) at a concentration of 0.3 µM. Bacteria were lysed according to published procedures, and then immobilized on glutathione Sepharose beads (Pharmacia). Briefly, MDA-MB-435 cell lysates were applied to GST- PAR_1_ C-tail or GST control columns. After 2 h binding periods to the designated protein/s cell lysates to the columns, a washing step was performed. The washes (×3) were carried out using a “wash buffer” including: 100 mM NaCL, 20 mM EDTA, 10 mM Tris, pH 8.0 and 1% Triton x100. This step was performed in order to wash out all non-specific proteins, leaving the GST- PAR_1_ -C-tail column firmly bound to targeted cell lysate proteins. Next, elution of bound proteins was performed via the addition of gel “sample buffer” and appropriate boiling. The samples were run electrophoretically on SDS-PAGE gels, followed by immunoblotting with the indicated antibodies and ECL detection.

### GST-PH-Etk/Bmx

The PH domain in Etk/Bmx was bound to GST column as previously described[25].

### Purification of PAR_1_ C-tail fragments

PAR_1_ C-tail fragments were generated using a “thrombin cleavage capture kit” (Novagen, Madison, WI; Cat no. 69022-3). The enzyme used for the cleavage was biotinylated human thrombin. Briefly, the cleavage was performed according to the manufacturer instructions. Biotinylated thrombin was removed from the cleavage reaction using streptavidin agarose beads, and the cleaved peptides (e.g., *wt* PAR_1_ -C-tail and Y381A C-tail) were isolated and loaded on a GST-Etk-PH column. After incubation for 4 h the purified fragments were applied onto the GST-PH-Etk/Bmx column and detected following gel separation and western blotting analysis using anti- PAR_1_ antibodies (ATAP, Santa Cruz, CA).

### Flow Cytometry Analysis

To activate PAR_1_, thrombin (1 U/ml was added for 5 min. The cells were detached from the plates with 0.5 mM EDTA in 0.1 M sodium phosphate at pH 7.4 (Biological Industries), washed and re-suspended in PBS. The cells were analyzed by FACS after incubation for 60 min at 4°C with 10 µg/ml anti- PAR_1_ -wede-PE antibodies.

### Western blot and immunoprecipitation analysis

Cells were activated with agonist peptide TFLLRNPNDK for the indicated periods of time and solubilized in lysis buffer containing10 mM Tris-HCl, pH 7.4, 150 mM NaCl, 1 mM EDTA, 1% TritonX-100, and protease inhibitors (5 mg/ml aprotinin, 1 mM phenylmethylsulfonylfluoride, and 10 mg/ml leupeptin) at 4°C for 30 min. The cell lysates were subjected to centrifugation at 12,000 rpm at 4°C for 20 min. We used 400 µg of the supernatants with anti- PAR_1_ (ATAP, Santa Cruz, CA 1 µg), anti-HA (anti-HA sc-7392; Santa Cruz, CA), anti-Shc or Etk/Bmx antibodies (10 µg/ml). After overnight incubation, Protein A-Sepharose beads (Amersham Pharmacia Biotech, Buckinghamshire, UK) were added to the suspension (50 µl), which was rotated at 4°C for 1 h. The immunocomplexes were eluted and run electrophoretically on a 10% SDS-PAGE gel, followed by transfer to an Immobilon-P membrane (Millipore). Membranes were blocked and probed with 1 µg/ml amounts of the appropriate antibodies as follows: anti- PAR_1_ thrombin receptor mAb, (ATAP, from Santa Cruz, 1∶1000); anti-Shc (BD, 1∶2000); anti-Bmx (Transduction Laboratories, 1∶1000) or anti-PY (Upstate 4G10, 1∶2500), suspended in 3% BSA in 10 mM Tris-HCl, pH 7.5, 100 mM NaCl, and 0.5% Tween-20. After washes the blots were incubated with secondary antibodies conjugated to horseradish-peroxidase. Immunoreactive bands were detected by enhanced chemiluminescence (ECL). Membranes were stripped and incubated with anti-IP antibodies to ensure equal protein load.

Anti- PAR_1_ polyclonal antibodies were generated using two regions of the N-terminus portion R/SFFLRN by the synthetic peptides NH_2_-CLLRNPNDKYEPFWED-COOH and NH_2-_KSSPLQKQLPAFISC-COOH.

### Antibody array

A custom-made antibody array (hypromatrix) containing thirty antibodies against suspected proteins was prepared (see Fig. S2). The antibodies were immobilized on a membrane, each at a pre-determined position, and they retained their capabilities of recognizing and capturing antigens as well as antigen-associated proteins. MDA-MB-435 cells were activated for 10 min with thrombin (1 U/ml). Untreated or activated cells were lysed with Triton extraction solution: 15 mM Tris, pH 7.5, 120 mM NaCl, 25 mM KCl, 2 mM EGTA, 2 mM EDTA, 0.1 mM DTT, 0.5% Triton X-100, 10 µg/ml leupeptin and 0.5 mM PMSF. Protein extract was incubated on pre-blocked membrane for 2 h at room temperature. The antibody array was washed with TBST and incubated with biotinylated PAR_1_ antibody (ATAP) for 2 h at room temperature. The antibody array was washed again with TBST, and the membrane was incubated with HRP-conjugated streptavidin for 1 h. Protein-protein interactions were detected by ECL and exposure to X-ray film.

### Matrigel invasion assay

Blind-well chemotaxis chambers with 13-mm diameter filters were used for this assay. Polyvinylpyrrolidone-free polycarbonate filters, 8 mm pore size (Costar Scientific Co., Cambridge, MA), were coated with basement membrane Matrigel (25 µg/filter), as previously described [43]. Briefly, the Matrigel was diluted to the desired final concentration with cold distilled water, applied to the filters, and dried under a hood. Cells (2×10^5^) suspended in DMEM containing 0.1% bovine serum albumin were added to the upper chamber. Conditioned medium of 3T3 fibroblasts was applied as a chemo-attractant and placed in the lower compartment of the Boyden chamber. Cells were incubated for 18 h on filters at 37°C in 5% CO_2_. At the end of the incubation, cells on the upper surface of the filter were removed by wiping with a cotton swab. The filters were fixed and stained with DifQuick System (Dade Behring Inc., Newark, NJ). Ten fields were chosen from the lower surface of the filter and cells within each field were counted. The mean +/− SD of the ten fields was calculated for each filter. Each assay was performed in triplicate.

### MCF10A morphogenesis assay

The assay was performed as previously described (24). In brief, while the MCF10A cells were maintained in DMEM/F12 medium with 20% donor horse serum, the cells for spheroid assay (DMEM/F12 supplemented with 2% donor horse serum, 10 µg/ml insulin, 1 ng/ml cholera-toxin, 100 µg/ml hydrocortisone, 50 U/ml penicillin and 50 µg/ml streptomycin) were resuspended at a concentration of 10^5^ cells per 4.0 ml. Eight-chambered RS glass slides (Nalgene) were coated with 35 µl Matrigel per well and left to solidify for 15 min. The cells were mixed 1∶1 with assay medium containing 4% Matrigel and 10 ng/ml EGF, and 400 µl were added to each chamber of the Matrigel-coated eight-chambered slide. Assay medium containing SFLLRNPNDK PAR_1_ activation peptide and 5 ng/ml EGF was replaced every 4 days. The images were taken between days 8–12. In the representative experiment shown images were taken on day 10. The media and supplements were replaced every 4 days and thus, the activating peptide was added fresh to the medium every 4 days.

## Supporting Information

Figure S1 Surface expression of various *hPar1* constructs (e.g., deletion constructs and Y/A mutations) following ectopic insertion to MCF7 cells. A. PAR1 constructs (e.g., wt, deleted constructs and Y/A mutations). Schematic representation of *hPar1* deletion constructs derived from human PAR1 cDNA. Mutations of Y/A insertions of the functional relevant Y residues in PAR1 C-tail (e.g., Y_381_A *hPar1* and the double mutant Y_381_A & Y_383_A *hPar1*) whereby replacement of Y/A at positions 381 and 383 of PAR1 C-tail was performed. B. FACS (fluorescent activated cell sorter) analysis. Flow cytometric analysis of surface-expressed *wt hPar1*, *hPar1* deletion constructs, Y381A *hPar1* and the double mutant Y_381_A & Y_383_A *hPar1*. Constructs were transiently expressed in COS-1 cells and surface expression was determined by flow cytometry analysis using anti-PAR1 abs (WEDE-PE 2584, Immunotech), directed to detect cell surface levels of PAR1 (analyses performed on intact cells). Empty peak - represents the isotype control antibody alone; black peak - represents PAR1 antibody. C. Histograms representing the surface expression of wt, deleted and mutant *hPar1* constructs before and after activation. Surface expression levels of the various *hPar1* constructs (e.g., *wt hPar1*, truncated *hPar1*, *Y397Z hPar1*, Y_381_A *hPar1* and Y_381_A & Y_383_A *hPar1*) transfected into COS-1 cells were determined. The various COS-1 transfected cells were evaluated by FACS analysis before (open bars) and after (black bars) a 30-minute activation with thrombin. Similar results were obtained in MCF7 cells expressing the *hPar1* various constructs (data not shown). D. PAR1 expression levels in MCF7 cells transfected with various *hPar1* constructs. Western blot analysis of MCF7 cells expressing either empty vector (A) or *Y397Z hPar1* (B), truncated *hPar1* (C), the double mutant Y_381_A & Y_383_A *hPar1*(D), Y_381_A *hPar1* (E), as also *wt hPar1* (F). Levels of protein loading were evaluated by b-actin.
(4.60 MB TIF)

Figure S2 Antibody-array of protein-protein interactions and physical association between PAR1 and the signaling partner Etk/Bmx. A. Custom-made antibody array. Table lists antibodies embedded on membranes showing the orientation map to create the custom array, as described in Materials and Methods. B. Lysates of MDA-435 cells before (i) and after (ii)thrombin (1 U/ml, 15 min) activation were applied to the membranes. Specific PAR-1 binding to the array was detected via incubation with biotinylated anti-PAR1 antibodies.
(4.60 MB TIF)

Figure S3 The phosphorylation status of Etk/Bmx associated PAR1 following PAR1 activation. Ai. HEK-293 cells were transfected with either wt Etk/Bmx or inactive KQ kinase -Etk/Bmx. Lysates were immunoprecipitated with anti Bmx and western blotted with 4G10 abs to detect levels of phosphorylation. Western blot analysis shows the levels of either endogenous Etk/Bmx (lanes c & d) or ectopically enforced Etk/Bmx (a & b) as compared to a house keeping gene b-actin. Aii. Endogenous levels of Etk/Bmx in HEK-293 cells. Western blot analysis was performed in lysates of HEK-293 cells before (c,d) and after (a,b) transfection with Etk/Bmx constructs. The equal loading levels were determined by a house keeping b-actin protein levels. B. PAR1-Bmx association. MDA-435 cells were TFLLRNPNDK-activated. Lysates were co-immunoprecipitated with anti-PAR1 antibodies, and eluted proteins were detected with Bmx antibodies. A strong association between PAR1 and Etk/Bmx was observed as early as 1 minute after activation reaching maximal levels after 10 minutes.
(2.05 MB TIF)

Figure S4 Characterization of MCF7 clones of HA-tagged wt and mutants of hPar1 constructs. A. FACS analysis of MCF7 clones. Surface expression of HA-*wt hPar1* and HA-*hPar1*-7A, was determined by using anti-PAR1 abs (WEDE-PE 2584, Immunotech). Empty peak - represents the isotype control antibody alone; black peak - represents PAR1 antibody. B. Histogram representing surface levels of the constructs (e.g., HA-*wt hPar1* and HA-*hPar1* 7A) as determined by FACS analysis. C. Western blot analysis of MCF7 cells transfected with empty vector and representative MCF7 clones (e.g., HA-*wt hPar1* and HA-*hPar1*-7A). The protein levels are compared to a house keeping of b-actin protein levels.
(2.05 MB TIF)

Figure S5 Immuno histological staining of Etk/Bmx in sections of mouse mammary tumor xenografts generated following implantation of MCF7 cells expressing wt hPar1 and variant constructs. MCF7 cells expressing various hPar1 forms (e.g., *wt hPar1*, truncated *hPar1*, Y_397_Z *hPar1* and empty vector) were inoculated into the mammary fat pads of mice. After 45 days the tumors were excised and embedded with paraffine. Antibodies directed against Etk/Bmx (Transduction Laboratories; BD Biosciences, California) were applied on sections derived from each of the designated treatment. The right panel represents staining in the absence of anti Etk/Bmx antibodies and presence of a secondary antibody - only (for controls). As one can note, specific Etk/Bmx staining is observed in *wt hPar1* and particularly strong staining is noticed in *Y397Z hPar1* (Mag ×200). No staining is observed in either the truncated *hPar1* or empty vector sections. This staining is a representative experiment of three times staining experiments performed on these mice mammary xenograft sections.
(2.05 MB TIF)
