# Correction: Etk/Bmx Regulates Proteinase-Activated-Receptor1 (PAR_1_) in Breast Cancer Invasion: Signaling Partners, Hierarchy and Physiological Significance

**DOI:** 10.1371/annotation/7ed84180-ec3d-46c0-8c10-f7b830999f8d

**Published:** 2010-12-29

**Authors:** Irit Cohen, Myriam Maoz, Hagit Turm, Sorina Grisaru-Granovsky, Bella Maly, Beatrice Uziely, Einat Weiss, Rinat Abramovitch, Eithan Gross, Oded Barzilay, Yun Qiu, Rachel Bar-Shavit

The author noted multiple errors in the original reference section. Here are the updated, correct reference section:

Corrected references:

1. Vu, T. K., Hung, D. T., Wheaton, V. I., & Coughlin, S. R. (1991) Cell 64, 1057-1068

2. Coughlin, S. R. (2000) Nature 407, 258-264

3. Rickles FR, Edwards RL (1983) Activation of blood coagulation in cancer: Trousseau's syndrome revisited. Blood 62: 14-31.

4. Camerer E, Duong DN, Hamilton JR, Coughlin SR (2004) Combined deficiency of protease-activated receptor-4 and fibrinogen recapitulates the hemostatic defect but not the embryonic lethality of prothrombin deficiency. Blood 103: 152-154.

5. Palumbo JS, Kombrinck KW, Drew AF, Grimes TS, Kiser JH, et al. (2000) Fibrinogen is an important determinant of the metastatic potential of circulating tumor cells. Blood 96: 3302-3309.

6. Boire A, Covic L, Agarwal A, Jacques S, Sherifi S, et al. (2005) PAR1 is a matrix metalloprotease-1 receptor that promotes invasion and tumorigenesis of breast cancer cells. Cell 120: 303-313.

7. Even-Ram S, Uziely B, Cohen P, Grisaru-Granovsky S, Maoz M, et al. (1998) Thrombin receptor overexpression in malignant and physiological invasion processes. Nat Med 4: 909-914.

8. Grisaru-Granovsky S, Salah Z, Maoz M, Pruss D, Beller U, et al. (2005) Differential expression of protease activated receptor 1 (Par1) and pY397FAK in benign and malignant human ovarian tissue samples. Int J Cancer 113: 372-378.

9. Even-Ram SC, Grisaru-Granovsky S, Pruss D, Maoz M, Salah Z, et al. (2003) The pattern of expression of protease-activated receptors (PARs) during early trophoblast development. J Pathol 200: 47-52.

10. Trejo J, Coughlin SR (1999) The cytoplasmic tails of protease-activated receptor-1 and substance P receptor specify sorting to lysosomes versus recycling. J Biol Chem 274: 2216-2224.

11. Hein L, Ishii K, Coughlin SR, Kobilka BK (1994) Intracellular targeting and trafficking of thrombin receptors. A novel mechanism for resensitization of a G protein-coupled receptor. J Biol Chem 269: 27719-27726.

12. Booden MA, Eckert LB, Der CJ, Trejo J (2004) Persistent signaling by dysregulated thrombin receptor trafficking promotes breast carcinoma cell invasion. Mol Cell Biol 24: 1990-1999.

13. Shapiro MJ, Trejo J, Zeng D, Coughlin SR (1996) Role of the thrombin receptor's cytoplasmic tail in intracellular trafficking. Distinct determinants for agonist-triggered versus tonic internalization and intracellular localization. J Biol Chem 271: 32874-32880.

14. Hammes SR, Coughlin SR (1999) Protease-activated receptor-1 can mediate responses to SFLLRN in thrombin-desensitized cells: evidence for a novel mechanism for preventing or terminating signaling by PAR1's tethered ligand. Biochemistry 38: 2486-2493.

15. Griffin CT, Srinivasan Y, Zheng YW, Huang W, Coughlin SR (2001) A role for thrombin receptor signaling in endothelial cells during embryonic development. Science 293: 1666-1670.

16. Connolly AJ, Ishihara H, Kahn ML, Farese RV, Jr., Coughlin SR (1996) Role of the thrombin receptor in development and evidence for a second receptor. Nature 381: 516-519.

17. Pelicci G, Lanfrancone L, Grignani F, McGlade J, Cavallo F, et al. (1992) A novel transforming protein (SHC) with an SH2 domain is implicated in mitogenic signal transduction. Cell 70: 93-104.

18. Pronk GJ, McGlade J, Pelicci G, Pawson T, Bos JL (1993) Insulin-induced phosphorylation of the 46- and 52-kDa Shc proteins. J Biol Chem 268: 5748-5753.

19. Kuruppu D, Christophi C, Maeda H, O'Brien PE (2002) Changes in the microvascular architecture of colorectal liver metastases following the administration of SMANCS/lipiodol. J Surg Res 103: 47-54.

20. Qiu Y, Kung HJ (2000) Signaling network of the Btk family kinases. Oncogene 19: 5651-5661.

21. Qiu Y, Robinson D, Pretlow TG, Kung HJ (1998) Etk/Bmx, a tyrosine kinase with a pleckstrin-homology domain, is an effector of phosphatidylinositol 3'-kinase and is involved in interleukin 6-induced neuroendocrine differentiation of prostate cancer cells. Proc Natl Acad Sci U S A 95: 3644-3649.

22. Tsai YT, Su YH, Fang SS, Huang TN, Qiu Y, et al. (2000) Etk, a Btk family tyrosine kinase, mediates cellular transformation by linking Src to STAT3 activation. Mol Cell Biol 20: 2043-2054.

23. Yin YJ, Salah Z, Grisaru-Granovsky S, Cohen I, Even-Ram SC, et al. (2003) Human protease-activated receptor 1 expression in malignant epithelia: a role in invasiveness. Arterioscler Thromb Vasc Biol. pp. 940-944.

24. Debnath J, Muthuswamy SK, Brugge JS. Morphogenesis and oncogenesis of MCF-10A mammary epithelial acini grown in three-dimensional basement membrane cultures. Methods. 2003 Jul;30(3):256-68

25. Karpatkin, S. (2007). Growth regulated oncogene is pivotal in thrombin-induced angiogenesis. Thromb Res 120 Suppl 2, S71-74

26. Yin YJ, Salah Z, Maoz M, Ram SC, Ochayon S, Neufeld G, et al. Oncogenic transformation induces tumor angiogenesis: a role for PAR1 activation. Faseb J 2003; 17: 163-74

27. Salah Z, Maoz M, Pokroy E, Lotem M, Bar-Shavit R, et al. (2007) Protease-activated receptor-1 (hPar1), a survival factor eliciting tumor progression. Mol Cancer Res 5: 229-240.

28. Chen Y, Grall D, Salcini AE, Pelicci PG, Pouysségur J, Van Obberghen-Schilling E. Shc adaptor proteins are key transducers of mitogenic signaling mediated by the G protein-coupled thrombin receptor. EMBO J. 1996 Mar 1;15(5):1037-44

29. Obreztchikova M, Elouardighi H, Ho M, Wilson BA, Gertsberg Z, et al. (2006) Distinct signaling functions for Shc isoforms in the heart. J Biol Chem 281: 20197-20204.

30. Collins LR, Ricketts WA, Olefsky JM, Brown JH (1997) The G12 coupled thrombin receptor stimulates mitogenesis through the Shc SH2 domain. Oncogene 15: 595-600.

31. Desgrosellier JS, Barnes LA, Shields DJ, Huang M, Lau SK, et al. (2009) An integrin alpha(v)beta(3)-c-Src oncogenic unit promotes anchorage-independence and tumor progression. Nat Med 15: 1163-1169.

32. Chen R, Kim O, Li M, Xiong X, Guan JL, et al. (2001) Regulation of the PH-domain-containing tyrosine kinase Etk by focal adhesion kinase through the FERM domain. Nat Cell Biol 3: 439-444.

33. Even-Ram SC, Maoz M, Pokroy E, Reich R, Katz BZ, et al. (2001) Tumor cell invasion is promoted by activation of protease activated receptor-1 in cooperation with the alpha vbeta 5 integrin. J Biol Chem 276: 10952-10962.

34. Abassi YA, Rehn M, Ekman N, Alitalo K, Vuori K (2003) p130Cas Couples the tyrosine kinase Bmx/Etk with regulation of the actin cytoskeleton and cell migration. J Biol Chem 278: 35636-35643.

35. Kim O, Yang J, Qiu Y (2002) Selective activation of small GTPase RhoA by tyrosine kinase Etk through its pleckstrin homology domain. J Biol Chem 277: 30066-30071.

36. Staal SP (1987) Molecular cloning of the akt oncogene and its human homologues AKT1 and AKT2: amplification of AKT1 in a primary human gastric adenocarcinoma. Proc Natl Acad Sci U S A 84: 5034-5037.

37. Paavonen K, Ekman N, Wirzenius M, Rajantie I, Poutanen M, et al. (2004) Bmx tyrosine kinase transgene induces skin hyperplasia, inflammatory angiogenesis, and accelerated wound healing. Mol Biol Cell 15: 4226-4233.

38. Lee LF, Guan J, Qiu Y, Kung HJ (2001) Neuropeptide-induced androgen independence in prostate cancer cells: roles of nonreceptor tyrosine kinases Etk/Bmx, Src, and focal adhesion kinase. Mol Cell Biol 21: 8385-8397.

39. Mao J, Xie W, Yuan H, Simon MI, Mano H, et al. (1998) Tec/Bmx non-receptor tyrosine kinases are involved in regulation of Rho and serum response factor by Galpha12/13. Embo J 17: 5638-5646.

40. Jiang Y, Ma W, Wan Y, Kozasa T, Hattori S, et al. (1998) The G protein G alpha12 stimulates Bruton's tyrosine kinase and a rasGAP through a conserved PH/BM domain. Nature 395: 808-813.

41. Ma YC, Huang XY (1998) Identification of the binding site for Gqalpha on its effector Bruton's tyrosine kinase. Proc Natl Acad Sci U S A 95: 12197-12201.

42. Bagheri-Yarmand R, Mandal M, Taludker AH, Wang RA, Vadlamudi RK, et al. (2001) Etk/Bmx tyrosine kinase activates Pak1 and regulates tumorigenicity of breast cancer cells. J Biol Chem 276: 29403-29409.

43. Swift S, Leger AJ, Talavera J, Zhang L, Bohm A, et al. (2006) Role of the PAR1 receptor 8th helix in signaling: the 7-8-1 receptor activation mechanism. J Biol Chem 281: 4109-4116.

44. Pan S, An P, Zhang R, He X, Yin G, et al. (2002) Etk/Bmx as a tumor necrosis factor receptor type 2-specific kinase: role in endothelial cell migration and angiogenesis. Mol Cell Biol 22: 7512-7523.

45. Arora P, Cuevas BD, Russo A, Johnson GL, Trejo J (2008) Persistent transactivation of EGFR and ErbB2/HER2 by protease-activated receptor-1 promotes breast carcinoma cell invasion. Oncogene 27: 4434-4445.

46. Groeger AM, Esposito V, De Luca A, Cassandro R, Tonini G, et al. (2004) Prognostic value of immunohistochemical expression of p53, bax, Bcl-2 and Bcl-xL in resected non-small-cell lung cancers. Histopathology 44: 54-63.

47. . Nakazato Y, Minami Y, Kobayashi H, Satomi K, Anami Y, et al. Nuclear grading of primary pulmonary adenocarcinomas: correlation between nuclear size and prognosis. Cancer 116: 2011-2019.

48. Dor Y, Djonov V, Abramovitch R, Itin A, Fishman GI, et al. (2002) Conditional switching of VEGF provides new insights into adult neovascularization and pro-angiogenic therapy. Embo J 21: 1939-1947.

49. Albini A, Iwamoto Y, Kleinman HK, Martin GR, Aaronson SA, et al. (1987) A rapid in vitro assay for quantitating the invasive potential of tumor cells. Cancer Res 47: 3239-3245.

