# Supplementary figures and images for: Etk/Bmx Regulates Proteinase-Activated-Receptor1 (PAR1) in Breast Cancer Invasion: Signaling Partners, Hierarchy and Physiological Significance
Source: PLoS One. 2010 Jun 15;5(6):e11135. doi: 10.1371/journal.pone.0011135 (PMC2886121; doi:10.1371/journal.pone.0011135)

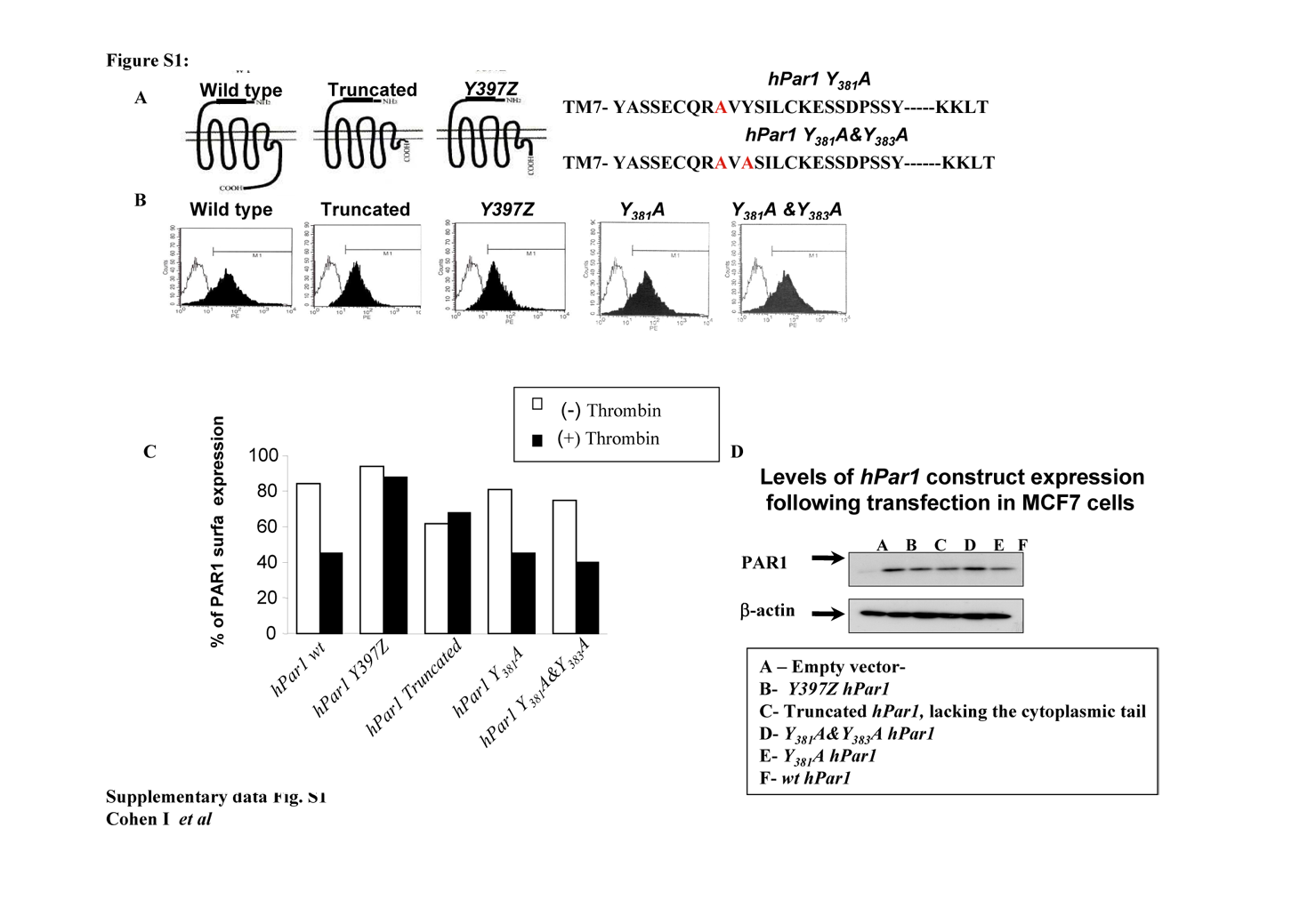

Supplement: Figure S1 — Surface expression of various hPar1 constructs (e.g., deletion constructs and Y/A mutations) following ectopic insertion to MCF7 cells. A. PAR1 constructs (e.g., wt, deleted constructs and Y/A mutations). Schematic representation of hPar1 deletion constructs derived from human PAR1 cDNA. Mutations of Y/A insertions of the functional relevant Y residues in PAR1 C-tail (e.g., Y381A hPar1 and the double mutant Y381A & Y383A hPar1) whereby replacement of Y/A at positions 381 and 383 of PAR1 C-tail was performed. B. FACS (fluorescent activated cell sorter) analysis. Flow cytometric analysis of surface-expressed wt hPar1, hPar1 deletion constructs, Y381A hPar1 and the double mutant Y381A & Y383A hPar1. Constructs were transiently expressed in COS-1 cells and surface expression was determined by flow cytometry analysis using anti-PAR1 abs (WEDE-PE 2584, Immunotech), directed to detect cell surface levels of PAR1 (analyses performed on intact cells). Empty peak - represents the isotype control antibody alone; black peak - represents PAR1 antibody. C. Histograms representing the surface expression of wt, deleted and mutant hPar1 constructs before and after activation. Surface expression levels of the various hPar1 constructs (e.g., wt hPar1, truncated hPar1, Y397Z hPar1, Y381A hPar1 and Y381A & Y383A hPar1) transfected into COS-1 cells were determined. The various COS-1 transfected cells were evaluated by FACS analysis before (open bars) and after (black bars) a 30-minute activation with thrombin. Similar results were obtained in MCF7 cells expressing the hPar1 various constructs (data not shown). D. PAR1 expression levels in MCF7 cells transfected with various hPar1 constructs. Western blot analysis of MCF7 cells expressing either empty vector (A) or Y397Z hPar1 (B), truncated hPar1 (C), the double mutant Y381A & Y383A hPar1(D), Y381A hPar1 (E), as also wt hPar1 (F). Levels of protein loading were evaluated by b-actin. (4.60 MB TIF) [file pone.0011135.s001.tif]

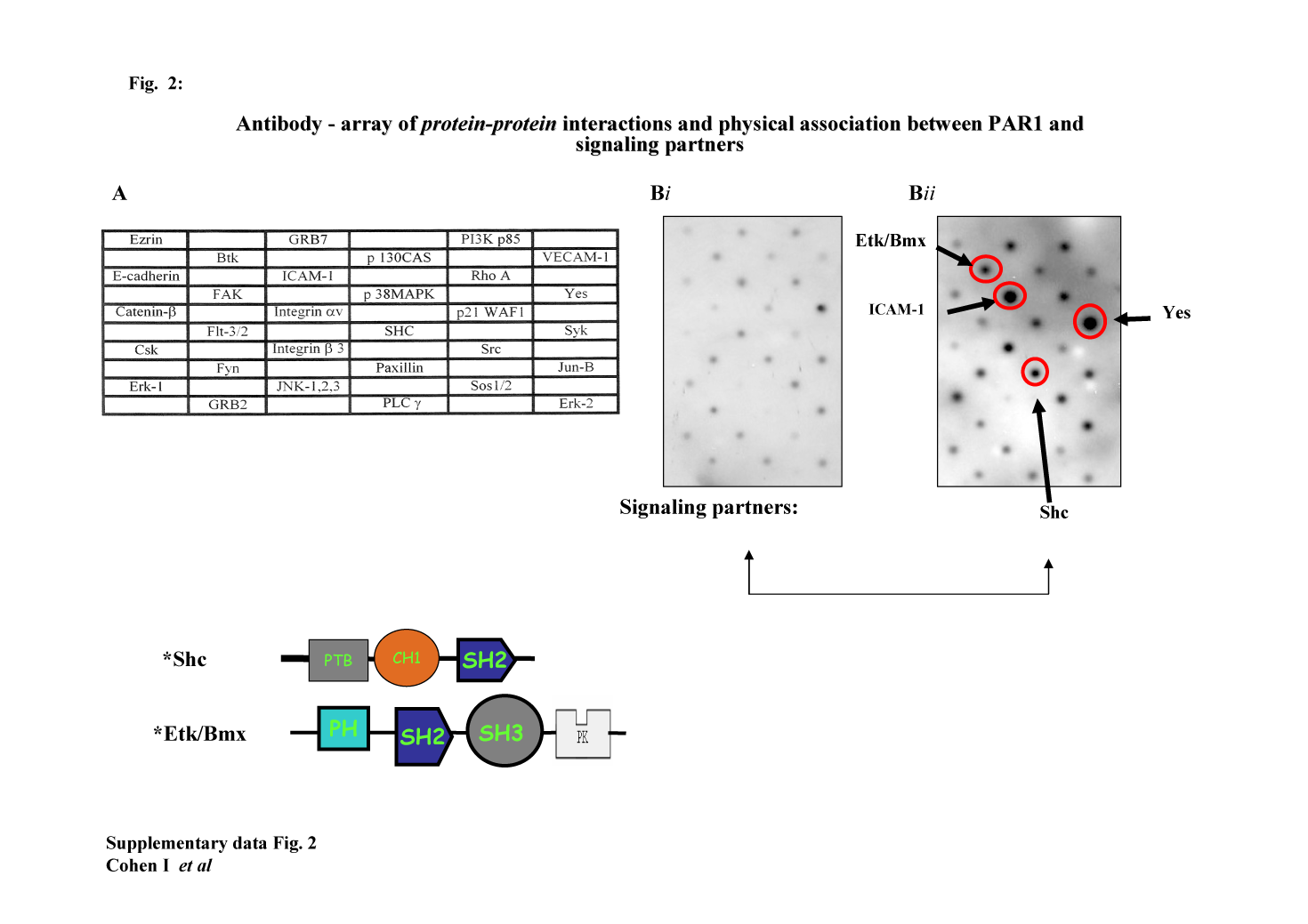

Supplement: Figure S2 — Antibody-array of protein-protein interactions and physical association between PAR1 and the signaling partner Etk/Bmx. A. Custom-made antibody array. Table lists antibodies embedded on membranes showing the orientation map to create the custom array, as described in Materials and Methods. B. Lysates of MDA-435 cells before (i) and after (ii)thrombin (1 U/ml, 15 min) activation were applied to the membranes. Specific PAR-1 binding to the array was detected via incubation with biotinylated anti-PAR1 antibodies. (4.60 MB TIF) [file pone.0011135.s002.tif]

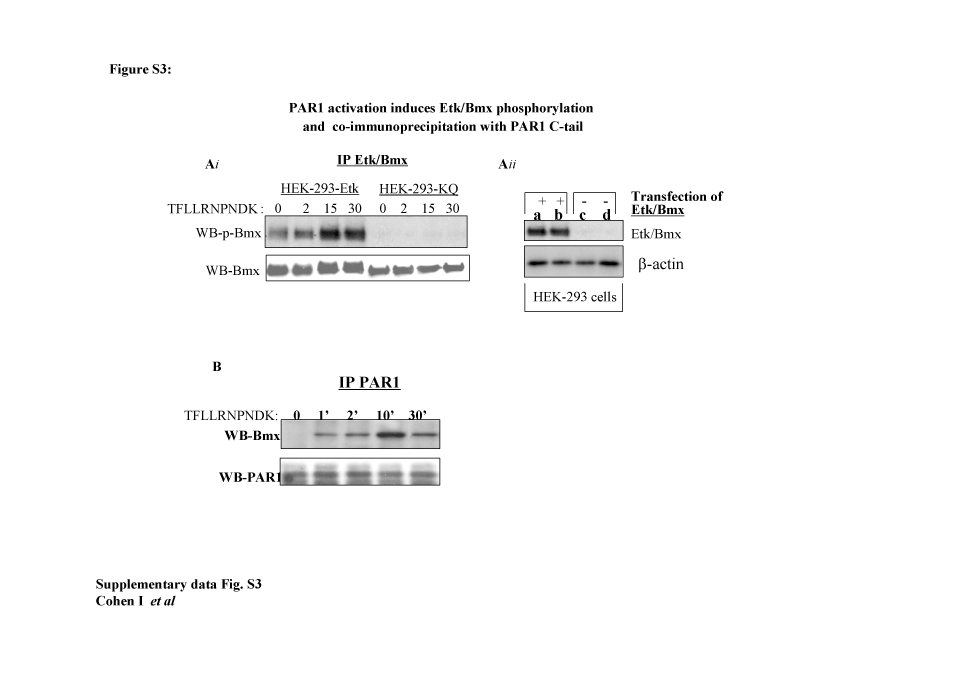

Supplement: Figure S3 — The phosphorylation status of Etk/Bmx associated PAR1 following PAR1 activation. Ai. HEK-293 cells were transfected with either wt Etk/Bmx or inactive KQ kinase -Etk/Bmx. Lysates were immunoprecipitated with anti Bmx and western blotted with 4G10 abs to detect levels of phosphorylation. Western blot analysis shows the levels of either endogenous Etk/Bmx (lanes c & d) or ectopically enforced Etk/Bmx (a & b) as compared to a house keeping gene b-actin. Aii. Endogenous levels of Etk/Bmx in HEK-293 cells. Western blot analysis was performed in lysates of HEK-293 cells before (c,d) and after (a,b) transfection with Etk/Bmx constructs. The equal loading levels were determined by a house keeping b-actin protein levels. B. PAR1-Bmx association. MDA-435 cells were TFLLRNPNDK-activated. Lysates were co-immunoprecipitated with anti-PAR1 antibodies, and eluted proteins were detected with Bmx antibodies. A strong association between PAR1 and Etk/Bmx was observed as early as 1 minute after activation reaching maximal levels after 10 minutes. (2.05 MB TIF) [file pone.0011135.s003.tif]

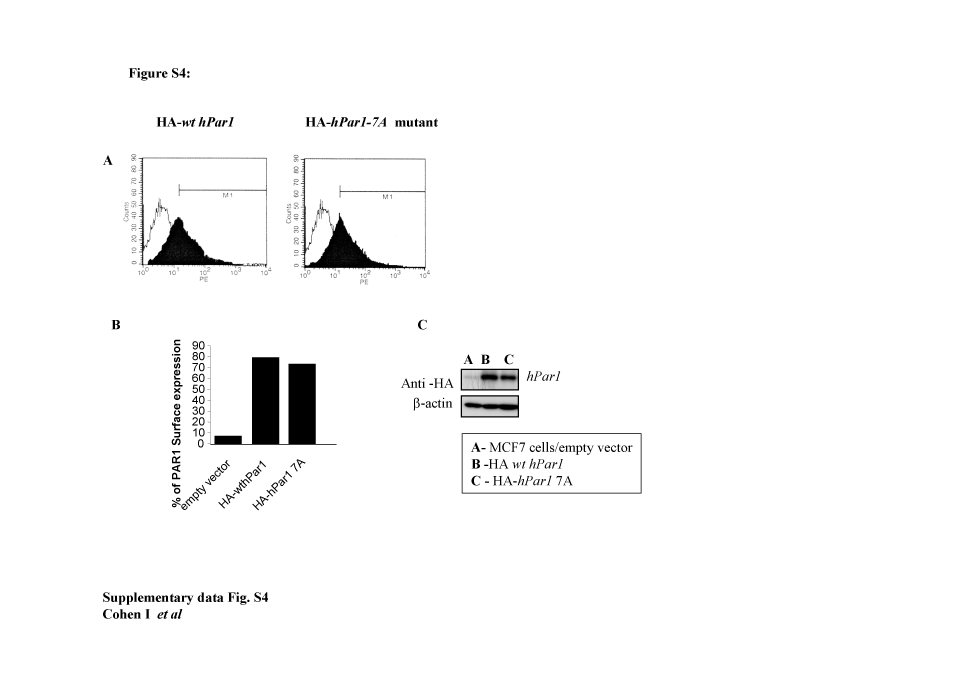

Supplement: Figure S4 — Characterization of MCF7 clones of HA-tagged wt and mutants of hPar1 constructs. A. FACS analysis of MCF7 clones. Surface expression of HA-wt hPar1 and HA-hPar1-7A, was determined by using anti-PAR1 abs (WEDE-PE 2584, Immunotech). Empty peak - represents the isotype control antibody alone; black peak - represents PAR1 antibody. B. Histogram representing surface levels of the constructs (e.g., HA-wt hPar1 and HA-hPar1 7A) as determined by FACS analysis. C. Western blot analysis of MCF7 cells transfected with empty vector and representative MCF7 clones (e.g., HA-wt hPar1 and HA-hPar1-7A). The protein levels are compared to a house keeping of b-actin protein levels. (2.05 MB TIF) [file pone.0011135.s004.tif]

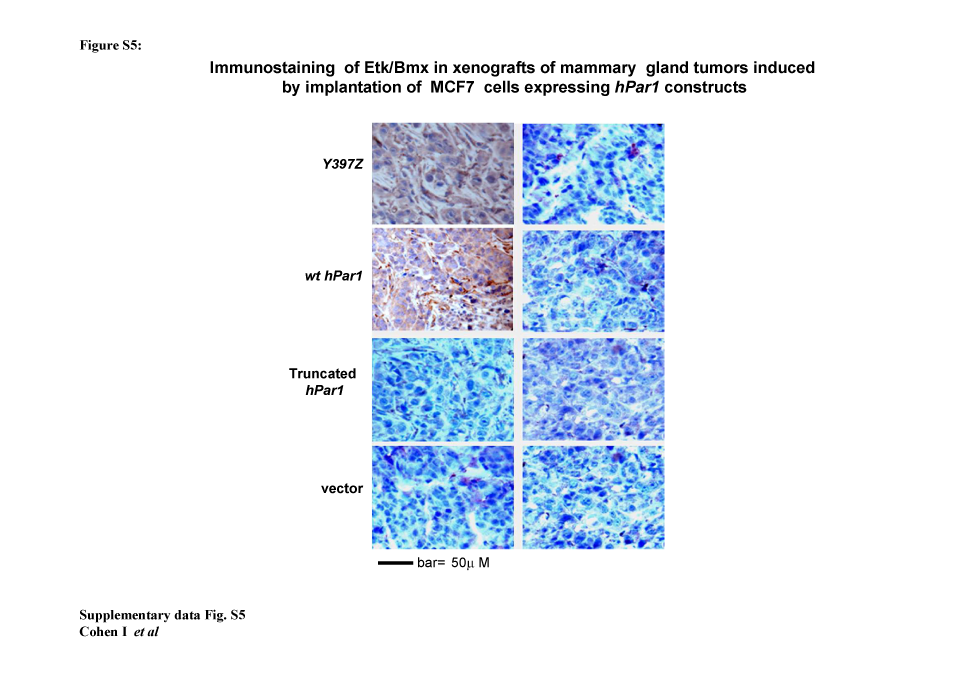

Supplement: Figure S5 — Immuno histological staining of Etk/Bmx in sections of mouse mammary tumor xenografts generated following implantation of MCF7 cells expressing wt hPar1 and variant constructs. MCF7 cells expressing various hPar1 forms (e.g., wt hPar1, truncated hPar1, Y397Z hPar1 and empty vector) were inoculated into the mammary fat pads of mice. After 45 days the tumors were excised and embedded with paraffine. Antibodies directed against Etk/Bmx (Transduction Laboratories; BD Biosciences, California) were applied on sections derived from each of the designated treatment. The right panel represents staining in the absence of anti Etk/Bmx antibodies and presence of a secondary antibody - only (for controls). As one can note, specific Etk/Bmx staining is observed in wt hPar1 and particularly strong staining is noticed in Y397Z hPar1 (Mag ×200). No staining is observed in either the truncated hPar1 or empty vector sections. This staining is a representative experiment of three times staining experiments performed on these mice mammary xenograft sections. (2.05 MB TIF) [file pone.0011135.s005.tif]
